# Global research trends and hotspots of short-chain fatty acids in cognitive impairment: a bibliometric analysis based on two databases

**DOI:** 10.3389/fmed.2026.1857769

**Published:** 2026-07-16

**Authors:** Xinru Wu, Tiancheng Yu, Shuai Hu, Tianyi Zhang

**Affiliations:** 1 Kunshan Integrated TCM and Western Medicine Hospital, Suzhou, China; 2 Physical Education and Sports School of Soochow University, Suzhou, China; 3 Institute of Physical Education, Jiangsu Normal University, Xuzhou, China; 4 Sports and Health Collaborative Innovation Center for Fitness Promotion, Jiangsu Normal University, Xuzhou, China

**Keywords:** Alzheimer’s disease, bibliometric analysis, cognitive impairment, gut microbiota, gut-brain axis, probiotics, short-chain fatty acids

## Abstract

**Objective:**

Short-chain fatty acids (SCFAs) have been widely investigated in research related to cognitive impairment, yet systematic bibliometric analyses focusing on their correlation remain relatively scarce. This study employed bibliometric analysis to objectively review relevant literature, identify key research contributors, and uncover emerging frontiers in the field.

**Methods:**

Relevant literature published from 2009 to 2025 was retrieved from the Web of Science Core Collection (WoSCC) and Scopus databases. Bibliometric analyses were performed using the Bibliometrix R package, VOSviewer, and CiteSpace software to evaluate research outputs and generate visualizations.

**Results:**

A total of 425 eligible articles from WoSCC and 514 from Scopus were included. Annual publications on SCFAs and cognitive impairment showed a continuous upward trend from 2009 to 2025. China contributed the largest number of publications in this field, and an international collaboration network has been established, with Wenzhou Medical University (China) serving as the core collaboration hub. International Journal of Molecular Sciences was identified as a major publication platform, and Zhang, Xin was recognized as a core author in SCFA-cognitive impairment research. High-frequency keywords included “gut microbiota,” “Alzheimer’s disease,” “neuroinflammation,” and “gut-brain axis.” In addition, recent research frontiers encompassed “drug therapy,” “microbiology,” and “chemistry,” revealing the core themes and trends of SCFA and cognitive impairment studies.

**Conclusion:**

This study conducted a comprehensive bibliometric analysis of the association between SCFAs and cognitive impairment, clarified the evolutionary trajectory of research themes, and identified potential future research directions. It revealed a developmental pattern whereby research in this field has gradually advanced from exploring basic correlations between diet and gut microbiota to in-depth investigations of pathological mechanisms, precise targeted interventions, and clinical translational applications. By systematically depicting the current research landscape, this study aims to provide guidance for subsequent investigations and fill critical knowledge gaps.

## 1 Introduction

Cognitive impairment is a brain dysfunction syndrome characterized by impairment in one or more cognitive domains, including memory, executive function, language, and visuospatial ability (1). It encompasses a continuous pathological spectrum ranging from mild cognitive impairment to dementia and has become one of the most severe public health challenges amid global population aging (2, 3). Core pathological alterations of the disease include neuronal loss (4), impaired synaptic plasticity (5), abnormal deposition of β-amyloid (6), hyperphosphorylation of tau protein, and chronic neuroinflammation (7, 8). Its onset and progression are driven jointly by genetic susceptibility and multiple environmental factors, with age, dietary patterns, lifestyle, metabolic disorders, and gut microbiota dysbiosis all confirmed as key risk factors (9–11). Epidemiological data indicate that approximately 55 million people worldwide lived with dementia in 2023, of whom more than 60% had dementia attributable to Alzheimer’s disease (AD) (12). Mild cognitive impairment (MCI), a high-risk prodromal stage of dementia, is characterized by mild cognitive deficits that do not meet the diagnostic criteria for dementia, with an annual conversion rate to dementia of 10–15%; the global prevalence of MCI has now exceeded 110 million individuals (13). Cognitive impairment is closely associated with numerous adverse outcomes, including physical dysfunction, increased fall risk, and elevated all-cause mortality (14), and is frequently accompanied by various neuropsychiatric symptoms such as anxiety, depression, and behavioral disturbances (15). This disease not only severely reduces patients’ quality of life but also imposes enormous pressure on global healthcare and public health systems (16). Importantly, effective disease-modifying treatments for moderate to severe cognitive impairment remain lacking. Therefore, exploring modifiable early targets has become a central research direction in this field.

In recent years, with the continuous refinement of the microbiota-gut-brain axis theory, accumulating studies have confirmed that the gut microbiota and its metabolites play a crucial regulatory role in the development and progression of cognitive impairment (17). Short-chain fatty acids (SCFAs) are major metabolites produced by anaerobic intestinal bacteria through the fermentation of indigestible carbohydrates such as dietary fiber, resistant starch, and oligosaccharides (18). Acetate, propionate, and butyrate are the primary components, serving as core messenger molecules mediating signal communication along the microbiota-gut-brain axis (19). Existing evidence suggests that SCFAs regulate the central nervous system microenvironment and cognitive function through multiple pathways, thereby influencing the pathological process of cognitive impairment (18). Animal and cellular experiments have revealed that SCFAs can specifically activate intestinal G protein-coupled receptors (GPCRs), stimulate intestinal L cells to secrete glucagon-like peptide-1 (GLP-1) and peptide YY (PYY), and regulate enterochromaffin cells to synthesize 5-hydroxytryptamine (5-HT) (20, 21). These effects further mediate microbiota-gut-brain axis signaling via the vagus nerve pathway, systemic circulation, and immune modulation, promoting hippocampal neurogenesis, improving synaptic plasticity, and ultimately exerting cognitive protective effects (22). On the other hand, SCFAs maintain intestinal mucosal barrier integrity by upregulating the expression of tight junction proteins, effectively reducing the entry of intestinal proinflammatory substances such as lipopolysaccharide into the bloodstream and inhibiting systemic low-grade inflammation (23). They can also cross the blood-brain barrier to suppress excessive microglial activation and the secretion of pro-inflammatory cytokines, which alleviates central neuroinflammation, reduces aberrant β-amyloid deposition and tau hyperphosphorylation, and ultimately ameliorates the core pathological features underlying cognitive impairment (24, 25). In addition, SCFAs production lowers intestinal luminal pH, creating an acidic microenvironment unfavorable to the colonization and proliferation of pathogenic bacteria, while promoting the growth of beneficial bacteria such as Bifidobacterium and Lactobacillus, thus maintaining the diversity and homeostasis of the gut microbiota (26, 27). This helps to block microbiota-gut-brain axis dysfunction and cognitive impairment mediated by gut dysbiosis (28). The synergistic effects of these multiple pathways collectively illustrate the pivotal regulatory role of SCFAs in the pathogenesis of cognitive impairment and highlight their potential value as promising targets for early prevention and targeted intervention of cognitive impairment.

Research on SCFAs and cognitive impairment has increased annually. However, existing studies mostly focus on the molecular mechanisms by which SCFAs regulate cognitive function or clinical verification of single intervention effects. A systematic, quantitative, and panoramic analysis of the developmental context, global publication trends, core collaboration networks, research hotspot clustering, and thematic evolution in this field based on large-scale global literature data is still lacking. Bibliometrics is a research method that quantitatively analyzes the external and content characteristics of literature using mathematical and statistical approaches. It can reveal the knowledge structure, developmental patterns, and emerging frontiers of specific research fields through various analyses, including temporal trends of literature output, mapping of national/institutional/author collaboration networks, keyword co-occurrence and clustering analysis, and co-citation and burst detection of references (29). This method has been widely applied in multiple medical disciplines such as neuroscience and geriatrics to identify mainstream research trends, detect knowledge gaps, and provide scientific guidance for future investigations. Therefore, this study combined bibliometric methods and visual analysis tools to conduct a systematic and comprehensive quantitative analysis of all literature related to SCFAs and cognitive impairment published between 2009 and 2025 in the Web of Science Core Collection and Scopus databases. We aimed to clarify the global developmental history and knowledge framework of this field, identify core research forces, mainstream research hotspots, and emerging frontier directions, and provide a macroscopic panoramic perspective and reliable empirical reference for researchers in this field to conduct follow-up studies.

## 2 Materials and methods

### 2.1 Literature sources and search strategy

Literature searches were performed in the WoSCC and Scopus databases on March 29, 2026. The search strategy for WoSCC was: TS = (“short-chain fatty acid*” OR “short chain fatty acid*” OR “fatty acids, volatile*” OR “volatile fatty acid*” OR “SCFA*” OR “acetate*” OR “propionate*” OR “butyrate*” OR “sodium butyrate*” OR “sodium acetate*” OR “sodium propionate*”) AND TS = (“cognitive dysfunction*” OR “cognitive impairment*” OR “neurocognitive disorder*” OR “cognitive decline*” OR “mild cognitive impairment” OR “dementia*” OR “vascular dementia*”) AND PY = (2009–2025) AND DT = (Article OR Review) AND LA = (English). Duplicate records were removed by matching titles or digital object identifiers, and irrelevant studies were excluded after screening. Finally, 425 eligible articles from WoSCC were included. All search records were saved in plain text format, and complete records including references were exported.

In the Scopus database, the search strategy was: TITLE-ABS-KEY (“short-chain fatty acid” OR “short chain fatty acid” OR “fatty acids, volatile” OR “volatile fatty acid” OR “SCFA” OR “acetate” OR “propionate” OR “butyrate” OR “sodium butyrate” OR “sodium acetate” OR “sodium propionate”) AND TITLE-ABS-KEY (“cognitive dysfunction” OR “cognitive impairment” OR “neurocognitive disorder” OR “cognitive decline” OR “mild cognitive impairment” OR “dementia” OR “vascular dementia”) AND PUBYEAR > 2000 AND PUBYEAR < 2026 AND (LIMIT-TO (DOCTYPE, “ar”) OR LIMIT-TO (DOCTYPE, “re”)) AND (LIMIT-TO (LANGUAGE, “English”)). Duplicate records were removed by matching titles or digital object identifiers, and irrelevant studies were excluded after screening. Finally, 514 eligible articles from Scopus were included. All Scopus records were saved in CSV format, and complete records including references were exported.

### 2.2 Inclusion and exclusion criteria

**FIGURE 1:**
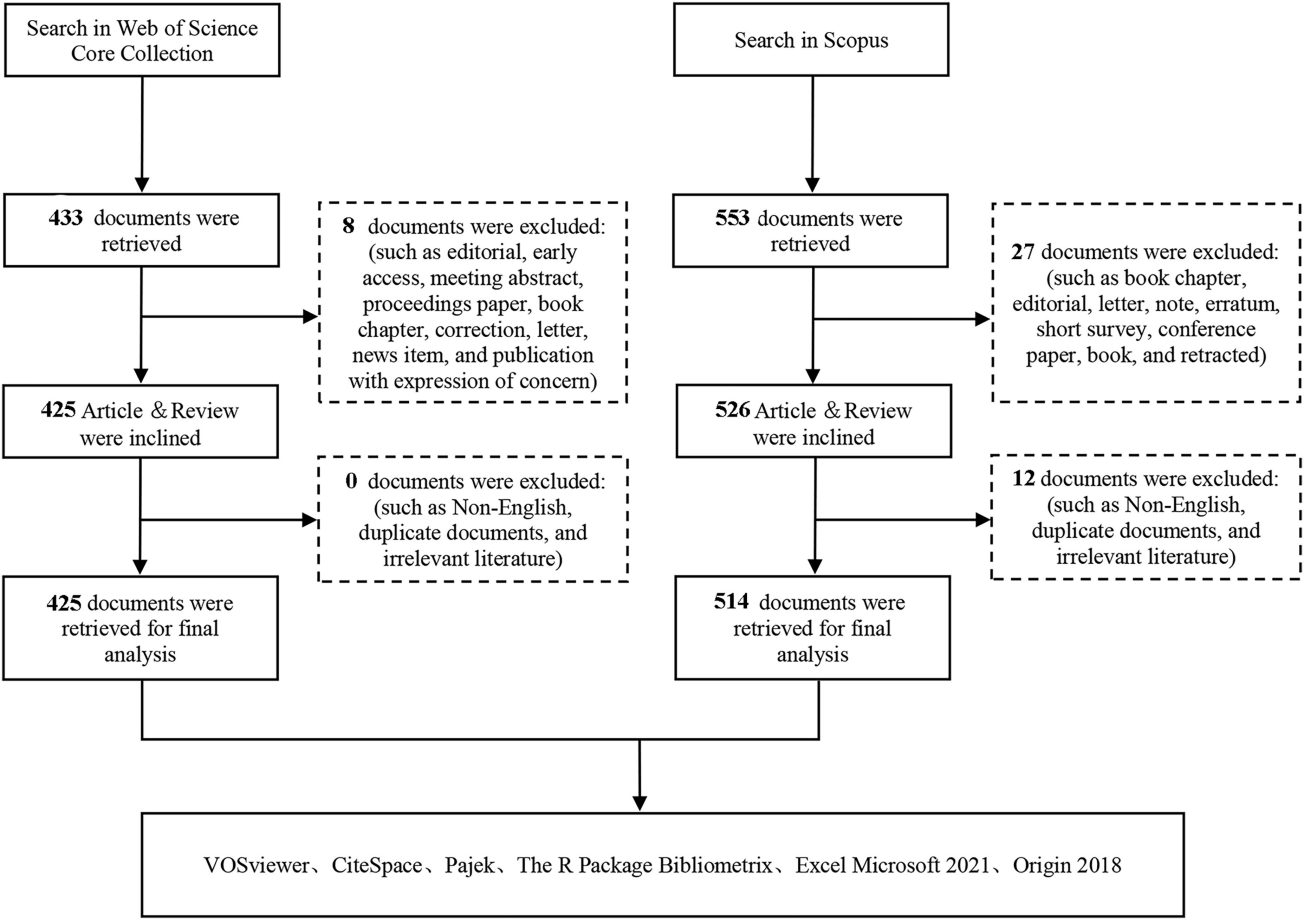
Flowchart of the publications selection.

The predefined inclusion criteria were as follows: English original research articles and reviews investigating the correlation between SCFAs and cognitive impairment. The study covered the full pathological spectrum of cognitive impairment, including multiple subtypes such as mild cognitive impairment, Alzheimer’s disease, and vascular dementia. The exclusion criteria comprised duplicate publications; literature irrelevant to the core themes of SCFAs and cognitive impairment; conference abstracts, conference proceedings, advance online publications, editorials, book chapters, retracted papers, and letters to the editor. To ensure data accuracy and the reproducibility of research results, all data extraction procedures were independently conducted by two researchers. The complete workflow of literature retrieval and screening is presented in Figure 1.

### 2.3 Data analysis

This study adopted a well-established and standardized bibliometric analytical framework. Origin 2018 was used to analyze annual publication trends. Meanwhile, R software (version 4.5.1) combined with the Bibliometrix package (version 4.01), VOSviewer (version 1.6.20), and CiteSpace (version 6.1.4) was applied to conduct multi-dimensional bibliometric analyses. Specifically, the Bibliometrix package was utilized for scientific knowledge mapping and visual analysis, whereas VOSviewer was employed to construct co-authorship networks at the national and institutional levels, visualize document co-citation relationships, and generate keyword co-occurrence maps.

In the co-authorship network analyses based on the WoSCC and Scopus datasets, the minimum publication threshold for countries and institutions was set to ≥ 5 articles to balance the integrity of network information and result interpretability. This threshold filters out entities with sporadic publications while retaining countries and institutions with sustained research output. We performed sensitivity analyses using two alternative thresholds of ≥ 3 and ≥ 10 publications. The results revealed that the overall network topology, core collaboration clusters, and leading countries and institutions remained highly consistent, with only minor fluctuations in peripheral nodes, demonstrating that the core collaboration patterns were robust to threshold adjustments. For co-citation analysis, the minimum citation frequency threshold was defined as ≥ 20. Sensitivity tests with thresholds of ≥ 15 and ≥ 30 citations showed stable core reference clusters and disciplinary knowledge structures, with only marginal changes observed among low-citation references, which verified the reliability of the selected threshold. A minimum occurrence threshold of ≥ 5 was adopted for keyword co-occurrence analysis to identify stable and representative research themes. Supplementary comparative analyses with thresholds of ≥ 3 and ≥ 7 occurrences yielded largely consistent research hotspots and thematic frameworks, with discrepancies limited to low-frequency peripheral keywords. CiteSpace was used to identify the top 25 most highly cited references in this field. The parameter settings were as follows: time slice range 2009–2025, time slice length 1 year, node type set to cited references, selection criterion set to top *N* = 50, and no pruning performed. Journal impact factor (IF) data used in this study were obtained from the 2024 Journal Citation Reports (JCR). Basic keywords such as “short-chain fatty acids,” “cognitive impairment” and their synonyms were excluded from the analysis.

## 3 Results

### 3.1 General overview of SCFAs and cognitive impairment

**FIGURE 2:**
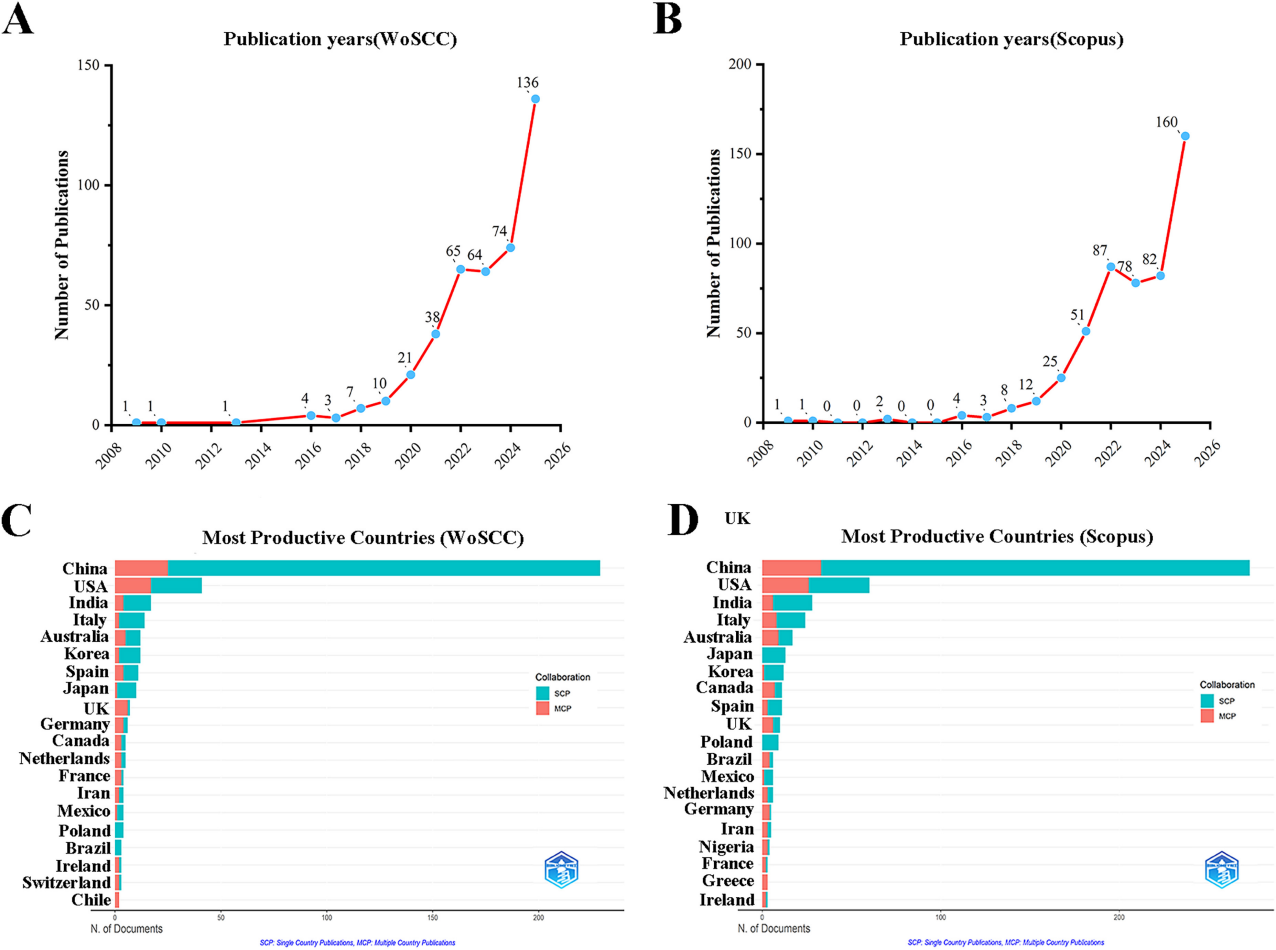
Annual publication trends on the relationship between SCFAs and cognitive impairment from 2009 to 2025. **(A)** Annual publication trends in WoSCC. **(B)** Annual publication trends in Scopus. **(C)** Distribution of corresponding authors’ countries in WoSCC. **(D)** Distribution of corresponding authors’ countries in Scopus.

A total of 425 independent publications were retrieved from the WoSCC database. The annual number of publications in this field showed an overall upward trend from 2009 to 2025, with a rapid increase especially after 2019, reaching a peak in 2025 (*n* = 136). A total of 514 deduplicated independent records were obtained from the Scopus database, and the publication trend was consistent with that of the WoSCC database, indicating continuously growing research interest in the association between SCFAs and cognitive impairment (Figures 2A,B).

**FIGURE 3:**
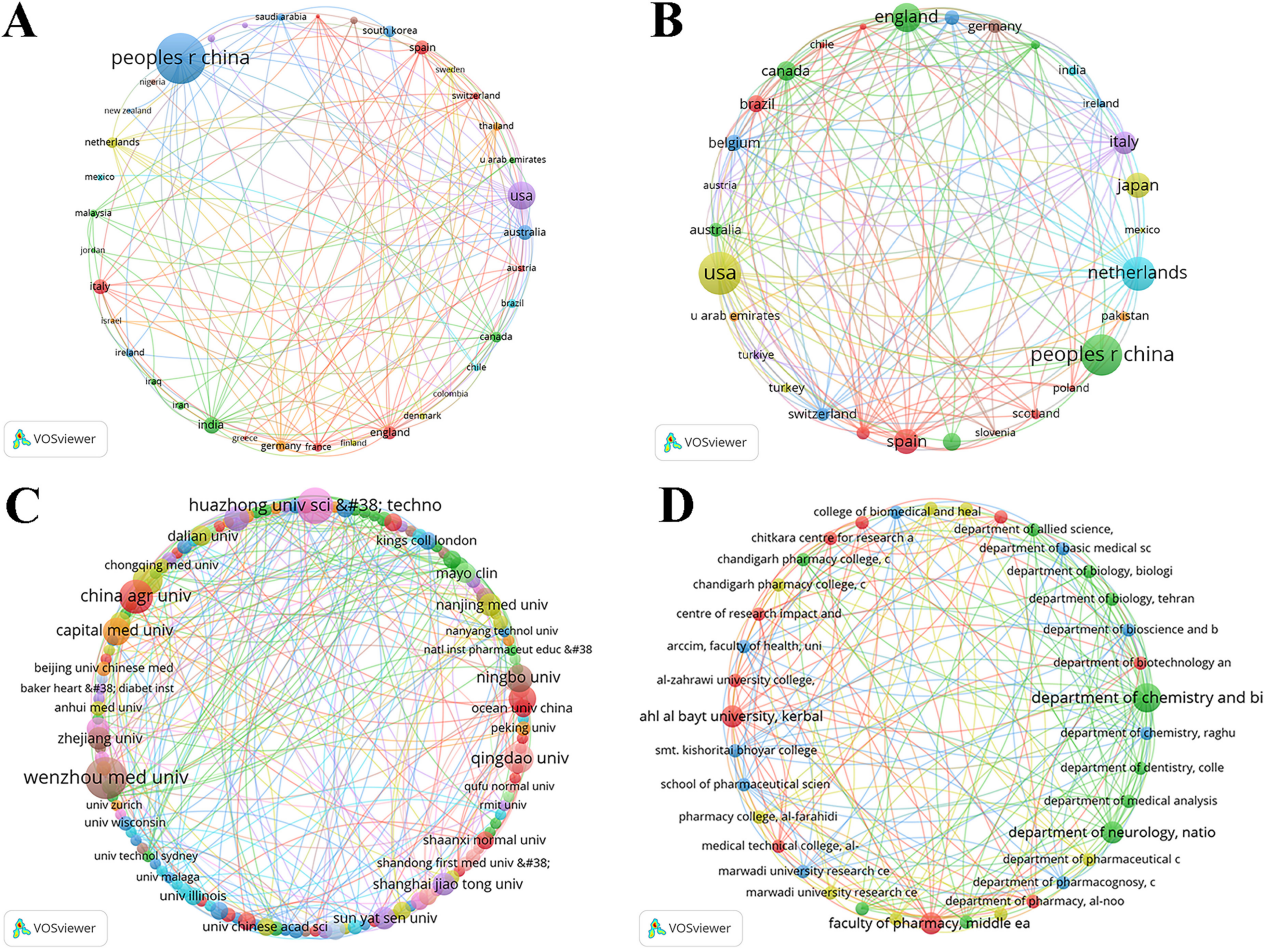
Global mapping of countries regions and institutions in SCFAs and cognitive impairment research from 2009 to 2025. **(A)** Geospatial collaboration network of countries based on WoSCC. **(B)** Geospatial collaboration network of countries based on Scopus. **(C)** Inter-institutional collaboration network derived from WoSCC. **(D)** Inter-institutional collaboration network derived from Scopus.

National contribution analysis revealed that in the WoSCC database, China ranked first in the number of publications (*n* = 229), followed by the United States (*n* = 41), India (*n* = 17), Italy (*n* = 14), and Australia (*n* = 12). Among them, 10.9% of publications from China and 41.5% from the US were international collaborative outputs (Figure 2C and Table 1). In the Scopus database, China still ranked first (*n* = 273), followed by the United States (*n* = 60), India (*n* = 28), Italy (*n* = 24), and Australia (*n* = 17). Similarly, 10.9% of publications from China and 41.5% from the US were collaborative studies involving multiple countries (Figure 2D and Table 1). In addition to leading publication output, China serves as the core hub of the international collaboration network and plays a key leading role in the development of this field (Figures 3A,B). Overall, China maintains a leading position in research on SCFAs and cognitive impairment, mainly supported by strategic investments such as the national brain initiative, a large aging population and domestic cohort resources, early deployment of microbiota-gut-brain axis research, collaboration among interdisciplinary teams and platforms, and explorations of precise mechanisms and interventions targeting the Chinese population, forming a complete research chain from basic science to clinical translation with global competitiveness.

**TABLE 1:** Most relevant countries by corresponding authors.

Country	Articles	SCP	MCP	Freq (%)	MCP_Ratio (%)
WoSCC					
China	229	204	25	53.9	10.9
USA	41	24	17	9.6	41.5
India	17	13	4	4	23.5
Italy	14	12	2	3.3	14.3
Australia	12	7	5	2.8	41.7
Korea	12	10	2	2.8	16.7
Spain	11	7	4	2.6	36.4
Japan	10	9	1	2.4	10
UK	7	1	6	1.6	85.7
Germany	6	2	4	1.4	66.7
Scopus					
China	273	240	33	48.1	12.1
USA	60	34	26	10.6	43.3
India	28	22	6	4.9	21.4
Italy	24	16	8	4.2	33.3
Australia	17	8	9	3	52.9
Japan	13	13	0	2.3	0
Korea	12	11	1	2.1	8.3
Canada	11	4	7	1.9	63.6
Spain	11	8	3	1.9	27.3
UK	10	4	6	1.8	60

MCP, Multiple country publication; SCP, Single country publication.

Institutional analysis further showed that in the WoSCC database, Wenzhou Medical University (China) contributed the largest number of publications (41), followed by the University of California System (United States, 26). In the Scopus database, the University of California (United States) ranked first (21), followed by Wenzhou Medical University (China, 19) (Table 2). Regarding collaboration networks, Wenzhou Medical University (China) acts as the central collaborative hub (Figures 3C,D). In summary, Wenzhou Medical University not only produces a high volume of publications but also maintains an extensive international collaboration network in this field.

**TABLE 2:** Top 10 most relevant affiliations of SCFAs and cognitive impairment.

WoSCC		Scopus	
Affiliation	Articles (n)	Affiliation	Articles (n)
Wenzhou Medical University	41	University of California	21
University Of California System	26	Wenzhou Medical University	19
University Of California San Diego	24	Huazhong University of Science and Technology	18
Huazhong University Of Science And Technology	22	University of Parma	17
Capital Medical University	21	Jiangnan University	15
Fudan University	20	University College Cork	14
Chinese Academy Of Sciences	18	Fudan University	13
National Taiwan University	17	Northwest Aandf University	13
University Of Parma	17	Capital Medical University	12
China Agricultural University	14	China Agricultural University	12

### 3.2 Journals and co-cited journals

**FIGURE 4:**
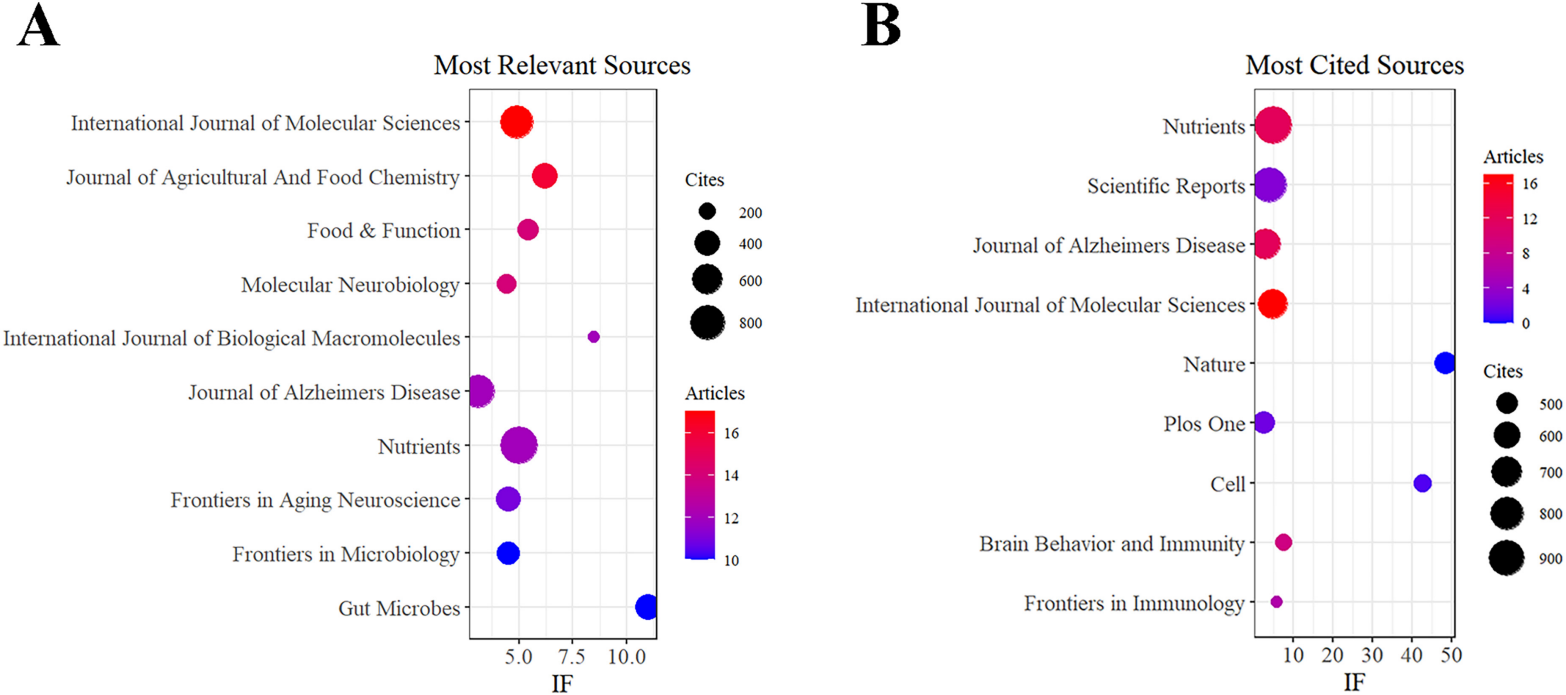
Journal with the largest number of articles published and the journal with the largest number of citations (WoSCC). **(A)** Journal with the largest number of articles published. **(B)** Journals with the largest number of citations.

To evaluate journal influence, this study used Bibliometrix for outcome analysis, ggplot2 for visualization, and VOSviewer to construct co-citation maps. The results showed that 425 publications from the WoSCC database were distributed across 372 academic journals (Table 3). As shown in Figure 4A, the journal with the highest number of publications was the *International Journal of Molecular Sciences* (*n* = 17, IF = 4.9), followed by the *Journal of Agricultural and Food Chemistry* (*n* = 16, IF = 6.2), *Food & Function* (*n* = 14, IF = 5.4), *Molecular Neurobiology* (*n* = 14, IF = 4.4), and the *International Journal of Biological Macromolecules* (*n* = 12, IF = 8.5). Figure 4B and Table 4 present the most frequently cited journals, including *Nutrients* (*n* = 957, IF = 5), *Scientific Reports* (*n* = 840, IF = 3.9), the *Journal of Alzheimer’s Disease* (*n* = 699, IF = 3.1), the *International Journal of Molecular Sciences* (n = 697, IF = 4.9), and *Nature* (*n* = 520, IF = 48.5).

**TABLE 3:** Top 10 journals with the most published articles.

WoSCC				Scopus		
Journal	Articles	Cites	IF (2024)	Journal	Articles	IF (2024)
International Journal of Molecular Sciences	17	697	4.9	International Journal of Molecular Sciences	23	4.9
Journal of Agricultural And Food Chemistry	16	387	6.2	Journal of Agricultural and Food Chemistry	19	6.2
Food and Function	14	276	5.4	Gut Microbes	13	11
Molecular Neurobiology	14	245	4.4	International Journal of Biological Macromolecules	13	8.5
International Journal of Biological Macromolecules	12	169	8.5	Nutrients	12	5
Journal of Alzheimer’s Disease	12	699	3.1	Brain, Behavior, And Immunity	10	7.1
Nutrients	12	957	5	Frontiers in Aging Neuroscience	10	4.5
Frontiers in Aging Neuroscience	11	352	4.5	Frontiers in Microbiology	10	4.5
Frontiers in Microbiology	10	313	4.5	Journal of Alzheimer’s Disease	10	3.1
Gut Microbes	10	388	11	Food and Function	9	5.4

**TABLE 4:** Top 10 journals with the most cited journals (WoSCC).

Rank	Journal	Cites	Articles	IF (2024)
1	Nutrients	957	12	5
2	Scientific Reports	840	3	3.9
3	Journal of Alzheimer’s Disease	699	12	3.1
4	International Journal of Molecular Sciences	697	17	4.9
5	Nature	520	0	48.5
6	Plos One	514	2	2.6
7	Cell	458	1	42.5
8	Brain Behavior and Immunity	452	9	7.6
9	Frontiers in Immunology	432	6	5.9
10	Nutrients	957	12	5

The 514 publications from the Scopus database were distributed across 241 academic journals (Table 3). The leading journal was the *International Journal of Molecular Sciences* (*n* = 17, IF = 4.9), followed by the *Journal of Agricultural and Food Chemistry* (*n* = 19, IF = 6.2), *Gut Microbes* (*n* = 13, IF = 11), the *International Journal of Biological Macromolecules* (*n* = 13, IF = 8.5), and *Nutrients* (*n* = 12, IF = 5).

**FIGURE 5:**
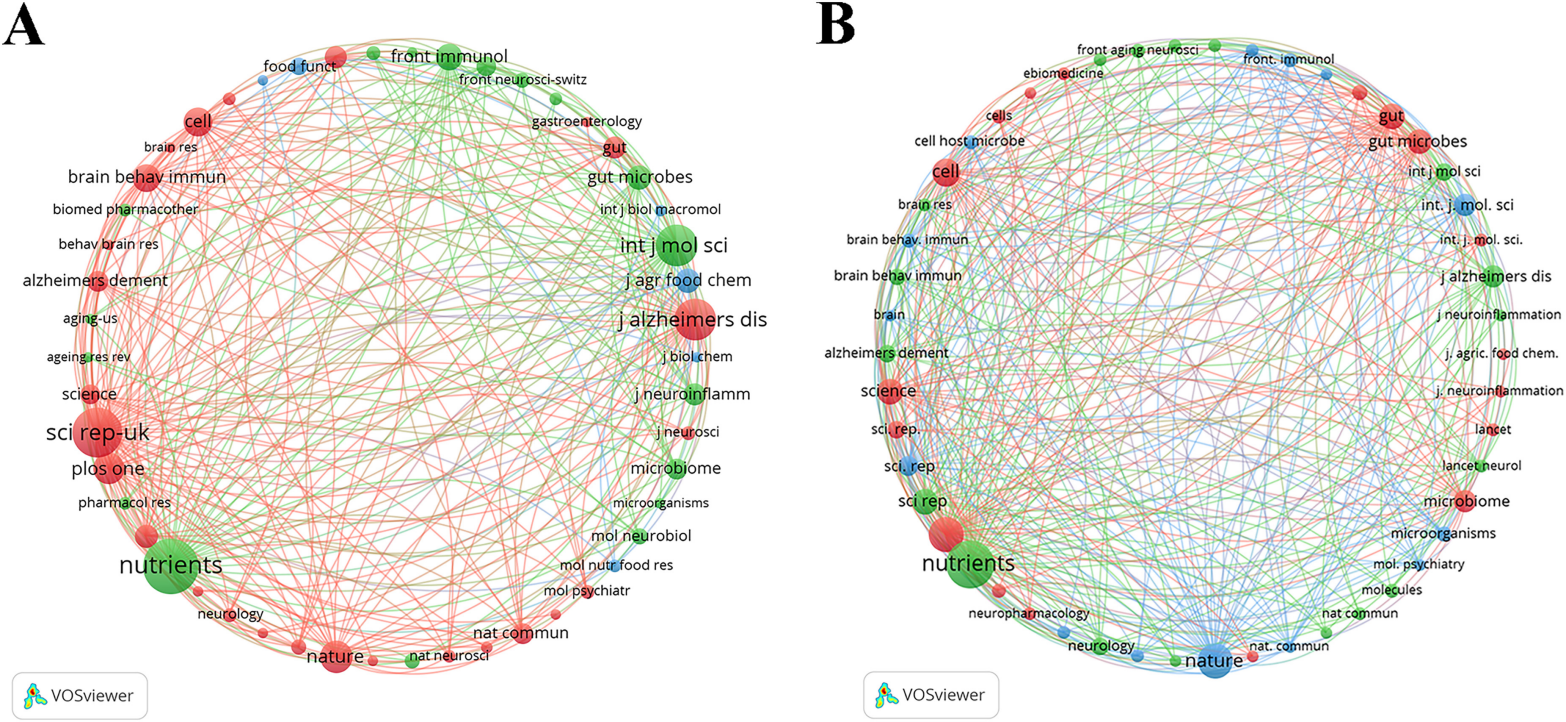
Co-cited journals related to SCFAs and cognitive impairment. **(A)** Journal co-citation network based on WoSCC. **(B)** Journal co-citation network based on Scopus.

Notably, the journal co-citation networks of both the WoSCC and Scopus databases (Figures 5A,B) revealed that *Nutrients* served as the central hub in the co-citation structure. These findings indicate that the *International Journal of Molecular Sciences* and *Nutrients* exert significant academic influence in the field of SCFAs and cognitive impairment research.

### 3.3 Author and co-author

Table 5 lists the top 10 authors by publication count in this field. In the WoSCC database, the top five authors were Zhang, Xin (*n* = 8), Liu, Jiaming (*n* = 5), Liu, Xuebo (*n* = 5), Liu, Zhigang (*n* = 5), and Sun, Jing (*n* = 5). Table 6 presents the top 10 authors by total citation frequency, with the top five being Gong, Tianyu (*n* = 68), Liu, Jiaming (*n* = 68), Sun, Jing (*n* = 68), Nagpal, Ravinder (*n* = 49), and Yadav, Hariom (*n* = 48).

**TABLE 5:** Top 10 documents authors related to SCFAs and cognitive impairment.

WoSCC			Scopus		
Authors	Documents	Citations	Authors	Documents	Citations
Zhang, Xin	8	1	Zhang, Xin	9	1
Liu, Jiaming	5	68	Liu, Xuebo	7	45
Liu, Xuebo	5	34	Liu, Zhigang	6	38
Liu, Zhigang	5	29	Cryan, John F.	5	39
Sun, Jing	5	68	Giridharan, Vijayasree V.	5	32
Bajaj, Jasmohan S.	4	3	Liu, Xia	5	32
Dai, Haochen	4	0	Liu, Zhou	5	13
Han, Xiaofei	4	4	Ticinesi, Andrea	5	9
Heo, Ho Jin	4	3	Bajaj, Jasmohan S	4	4
Kim, Hyun-Jin	4	3	Barichello, Tatiana	4	30

**TABLE 6:** Top 10 citations authors related to SCFAs and cognitive impairment (WoSCC).

Rank	Authors	Citations	Documents
1	Gong, Tianyu	68	3
2	Liu, Jiaming	68	5
3	Sun, Jing	68	5
4	Nagpal, Ravinder	49	4
5	Yadav, Hariom	48	4
6	Craft, Suzanne	47	2
7	Neth, Bryan J.	47	2
8	Wang, Shaohua	47	2
9	Ling, Yi	41	2
10	Chen, Danna	38	1

In the Scopus database, Zhang, Xin had the largest number of publications (*n* = 9), followed by Liu, Xuebo (*n* = 7), Liu, Zhigang (*n* = 6), Cryan, John F. (*n* = 5), and Giridharan, Vijayasree V. (n = 5). Among the top 10 authors by total citation frequency, the top five were Gong, Tianyu (*n* = 68), Liu, Jiaming (*n* = 68), Sun, Jing (*n* = 68), Nagpal, Ravinder (*n* = 49), and Yadav, Hariom (*n* = 49).

**FIGURE 6:**
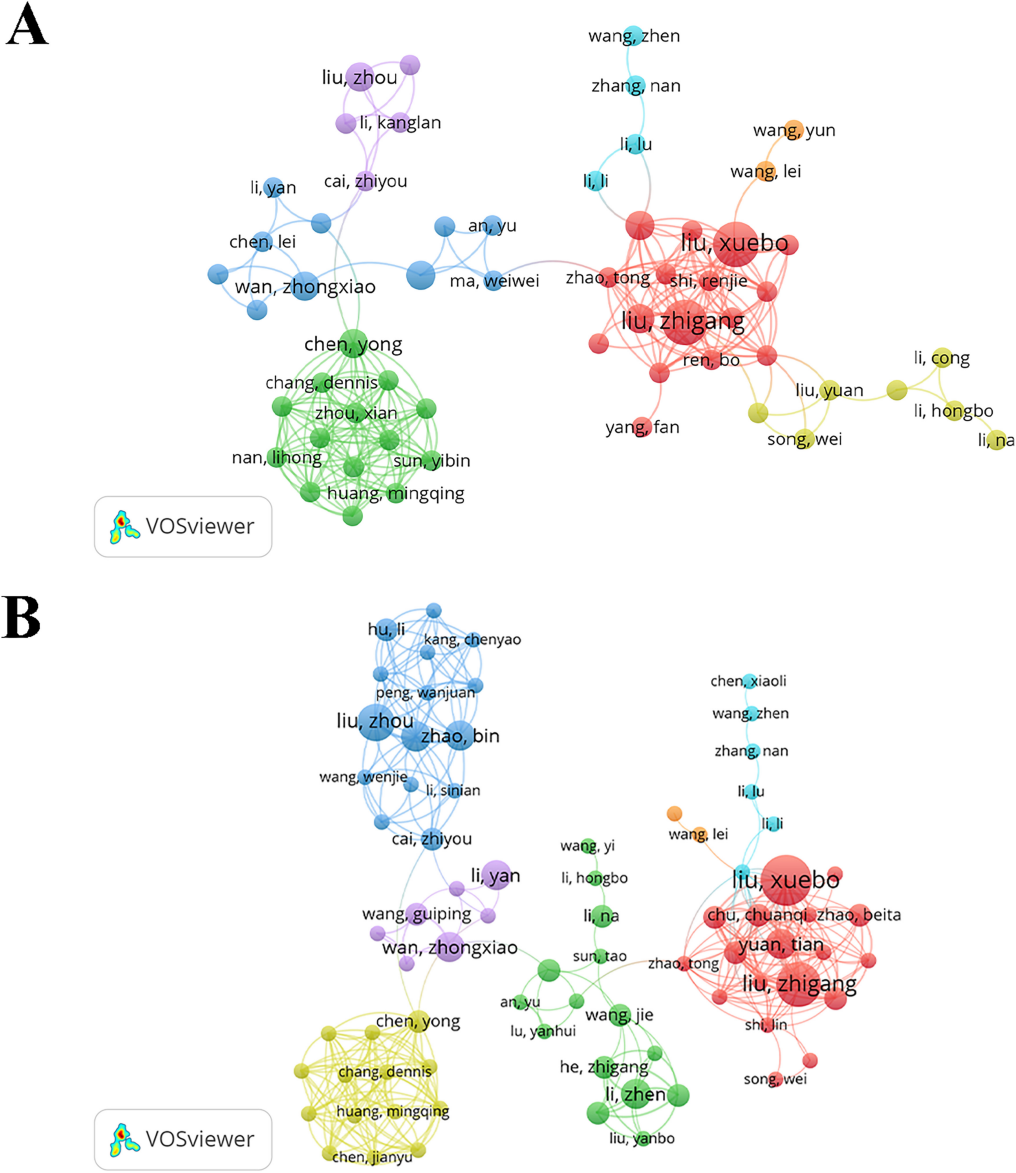
The map of co-authorship in the field of SCFAs and cognitive impairment. **(A)** Co-authorship network based on WoSCC. **(B)** Co-authorship network based on Scopus.

Author co-authorship network maps for the WoSCC and Scopus databases (Figure 6) showed extensive and close collaboration among researchers in this field, with Liu, Xuebo and Liu, Zhigang serving as core nodes in the collaboration network, indicating their profound influence on the field.

### 3.4 Most cited references and reference burst

In this study, the bibliometrix package in R software was used to systematically identify the top 25 highly cited articles in the field of short-chain fatty acids and cognitive impairment. In the WoSCC database, each of the top 25 highly cited papers was cited more than 110 times (Table 7). The top three most frequently cited articles were *The neuropharmacology of butyrate: The bread and butter of the microbiota-gut-brain axis?*, *Modified Mediterranean-ketogenic diet modulates gut microbiome and short-chain fatty acids in association with Alzheimer’s disease markers in subjects with mild cognitive impairment*, and *Gut microbiota mediates intermittent-fasting alleviation of diabetes-induced cognitive impairment*.

**TABLE 7:** Top 25 cited references related to SCFAs and cognitive impairment (WoSCC).

Paper	DOI	Total citations	TC per year
Stilling RM, 2016, Neurochem Int	10.1016/j.neuint.2016.06.011	693	63.00
Nagpal R, 2019, Ebiomedicine	10.1016/j.ebiom.2019.08.032	501	62.63
Liu ZG, 2020, Nat Commun	10.1038/s41467-020-14676-4	442	63.14
Sun J, 2019, Transl Psychiat	10.1038/s41398-019-0525-3	423	52.88
Chen RZ, 2019, Pharmacol Res	10.1016/j.phrs.2019.104403	329	41.13
Mirzaei R, 2021, Biomed Pharmacother	10.1016/j.biopha.2021.111661	315	52.50
Matt SM, 2018, Front Immunol	10.3389/fimmu.2018.01832	261	29.00
Marizzoni M, 2020, J Alzheimers Dis	10.3233/JAD-200306	257	36.71
Mitsou EK, 2017, Brit J Nutr	10.1017/S0007114517001593	248	24.80
Shi HL, 2021, Microbiome	10.1186/s40168-021-01172-0	215	35.83
Xiao WP, 2022, Microbiome	10.1186/s40168-022-01255-6	205	41.00
Chen SJ, 2022, Neurology	10.1212/WNL.0000000000013225	205	41.00
Peh A, 2022, Stroke	10.1161/STROKEAHA.121.036800	182	36.40
Verhaar BJH, 2022, Front Immunol	10.3389/fimmu.2021.794519	180	36.00
Maqsood R, 2016, Neurochem Res	10.1007/s11064-016-2039-1	179	16.27
Tran TTT, 2019, Faseb J	10.1096/fj.201900071R	177	22.13
Wu L, 2021, Nutrients	10.3390/nu13010228	173	28.83
wei HL, 2023, Biomed Pharmacother	10.1016/j.biopha.2023.114308	172	43.00
SUN JQ, 2016, NUTR J	10.1186/s12937-016-0147-z	166	15.09
Tan AH, 2021, Ann Neurol	10.1002/ana.25982	163	27.17
Liu JM, 2020, J Agr Food Chem	10.1021/acs.jafc.0c02807	162	23.14
Ren TZ, 2020, Front Neurol	10.3389/fneur.2020.00137	142	20.29
Generoso JS, 2021, Braz J Psychiat	10.1590/1516-4446-2020-0987	136	22.67
Nagpal R, 2020, Ebiomedicine	10.1016/j.ebiom.2020.102950	136	19.43
Orszaghova Z, 2021, Front Mol Biosci	10.3389/fmolb.2021.770413	133	22.17

In the Scopus database, each of the top 25 highly cited papers was cited more than 283 times (Table 8). The top three most frequently cited articles were *The neuropharmacology of butyrate: The bread and butter of the microbiota-gut-brain axis?*, *Gut Microbial Ecosystem in Parkinson Disease: New Clinicobiological Insights from Multi-Omics*, and *Modified Mediterranean-ketogenic diet modulates gut microbiome and short-chain fatty acids in association with Alzheimer’s disease markers in subjects with mild cognitive impairment*.

**TABLE 8:** Top 25 cited references related to SCFAs and cognitive impairment (Scopus).

Paper	DOI	Total citations	TC per year
Stilling RM, 2016, Neurochem Int	10.1016/j.neuint.2016.06.011	766	69.64
Loh JS, 2024, Signal Transduct Target Ther	10.1038/s41392-024-01743-1	745	248.33
Nagpal R, 2019, Ebiomedicine	10.1016/j.ebiom.2019.08.032	529	66.13
Liu Z, 2020, Nat Commun	10.1038/s41467-020-14676-4	498	71.14
Sun J, 2019, Transl Psychiatry	10.1038/s41398-019-0525-3	453	56.63
Diederich M, 2010, J Biomed Biotechnol	10.1155/2010/479364	433	25.47
Chen C, 2022, GUT	10.1136/gutjnl-2021-326269	382	76.40
Mirzaei R, 2021, Biomed Pharmacother	10.1016/j.biopha.2021.111661	341	56.83
Doifode T, 2021, Pharmacol Res	10.1016/j.phrs.2020.105314	293	48.83
Marizzoni M, 2020, J Alzheimer’s Dis	10.3233/JAD-200306	273	39.00
Noble EE, 2017, Front Behav Neurosci	10.3389/fnbeh.2017.00009	265	26.50
Alkasir R, 2017, Protein Cell	10.1007/s13238-016-0338-6	250	25.00
Chen S-J, 2022, Neurology	10.1212/WNL.0000000000013225	229	45.80
Xiao W, 2022, Microbiome	10.1186/s40168-022-01255-6	219	43.80
Shi H, 2021, Microbiome	10.1186/s40168-021-01172-0	218	36.33
Logsdon AF, 2018, Exp Biol Med	10.1177/1535370217743766	206	22.89
Maqsood R, 2016, Neurochem Res	10.1007/s11064-016-2039-1	206	18.73
Jianqin S, 2016, Nutr J	10.1186/s12937-016-0147-z	198	18.00
Tran TTT, 2019, Faseb J	10.1096/fj.201900071R	186	23.25
Wei H, 2023, Biomed Pharmacother	10.1016/j.biopha.2023.114308	185	46.25
Verhaar BJH, 2022, Front Immunol	10.3389/fimmu.2021.794519	185	37.00
Wu L, 2021, Nutrients	10.3390/nu13010228	184	30.67
Tan AH, 2021, Ann Neurol	10.1002/ana.25982	174	29.00
Liu J, 2020, J Agric Food Chem	10.1021/acs.jafc.0c02807	172	24.57
Chidambaram SB, 2022, Pharmacol Ther	10.1016/j.pharmthera.2021.107988	166	33.20

**FIGURE 7:**
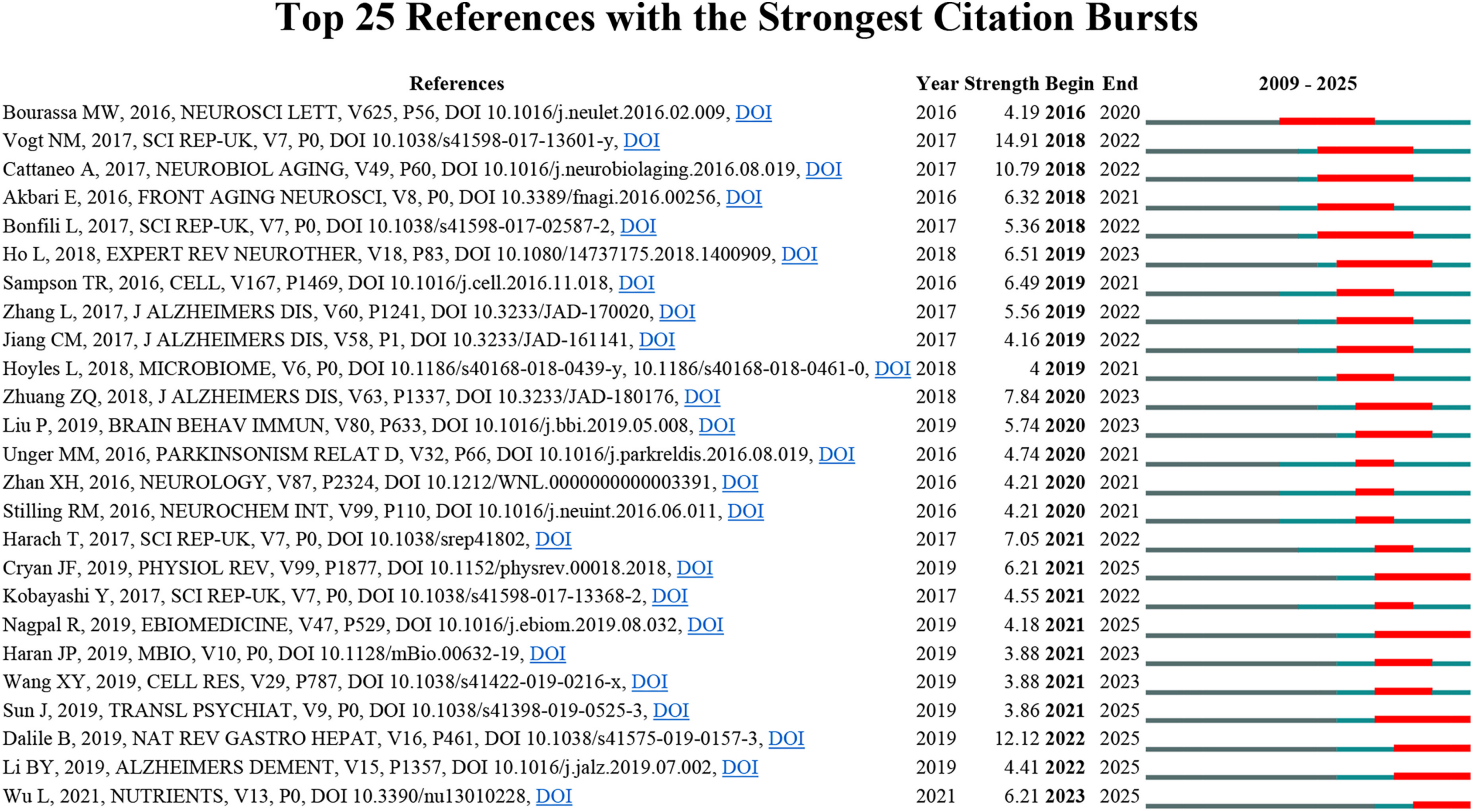
Top 25 references with the strongest citation bursts in SCFAs and cognitive impairment (WoSCC).

CiteSpace analysis revealed 25 articles with citation bursts in the WoSCC database (Figure 7). The three articles with the strongest citation bursts were *Gut microbiome alterations in Alzheimer’s disease* (burst strength = 14.91), *The role of short-chain fatty acids in microbiota-gut-brain communication* (burst strength = 12.12), and *Association of brain amyloidosis with pro-inflammatory gut bacterial taxa and peripheral inflammation markers in cognitively impaired elderly* (burst strength = 10.79).

Combined with the results of reference citation analysis and citation burst detection, current research in this field mainly focuses on: (1) The molecular and neuropharmacological mechanisms by which SCFAs mediate the regulation of cognitive impairment via the microbiota-gut-brain axis. (2) Clinical characteristics and biomarkers of gut microbiota-SCFA axis disturbance in AD and its prodromal stage, MCI. (3) Intervention strategies targeting the gut microbiota-SCFA axis to ameliorate cognitive impairment.

### 3.5 Keyword clusters and thematic evolution

**FIGURE 8:**
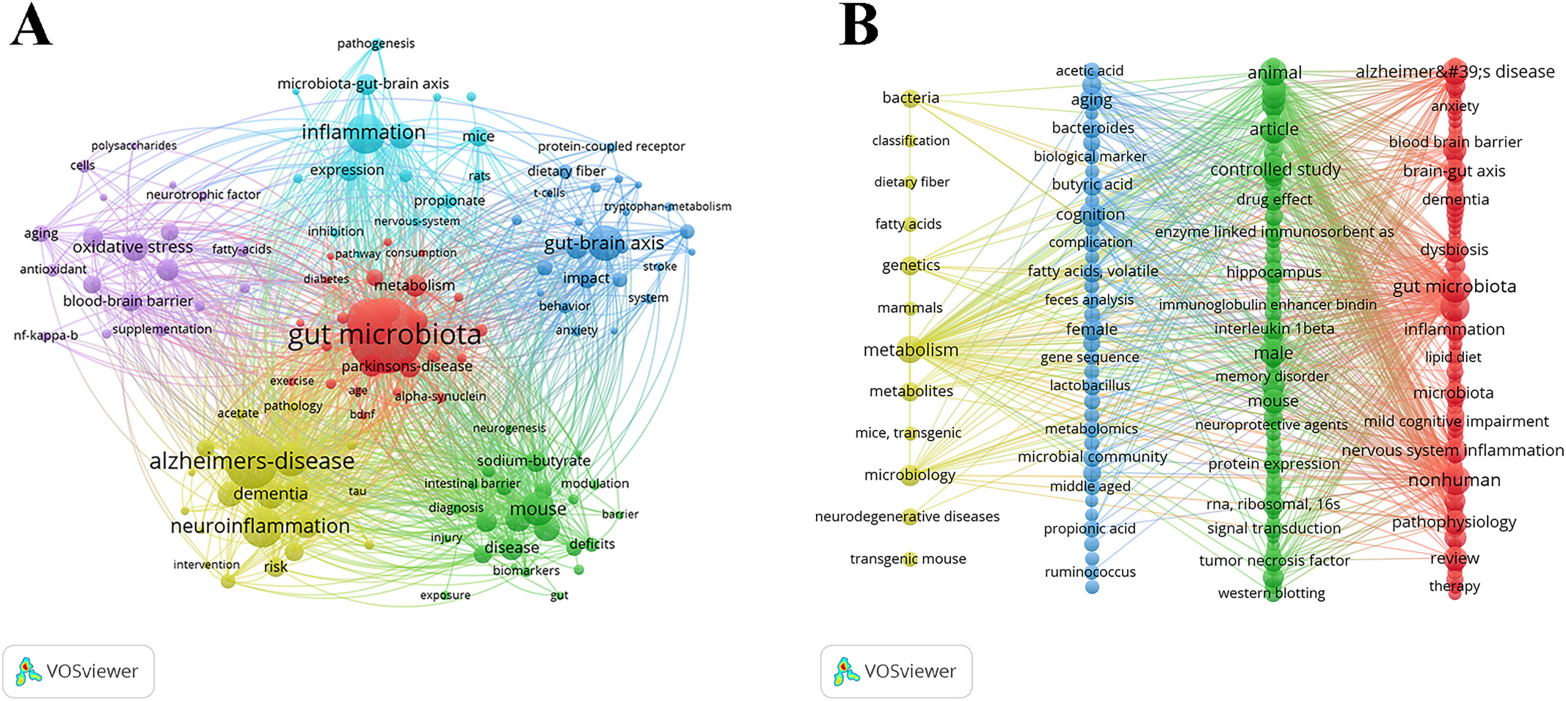
Keyword co-occurrence map of publications in SCFAs and cognitive impairment. **(A)** Keyword co-occurrence analysis based on WoSCC. **(B)** Keyword co-occurrence analysis based on Scopus.

High-frequency keywords can reflect the research frontiers and dynamics of a specific knowledge domain. Using VOSviewer software, a total of 1928 keywords were identified in the WoSCC database in this study. The top 10 high-frequency keywords listed in Table 9 all appeared ≥ 43 times, among which “gut microbiota” showed the highest frequency (*n* = 236), followed by “Alzheimer’s disease” (*n* = 130) and “microbiota” (*n* = 97). Subsequently, based on the screening criterion of keyword occurrence ≥ 5, 136 keywords were selected to construct a keyword clustering map (Figure 8), and six distinct color-coded clusters were observed. Red Cluster 1 focused on the metabolic mechanisms of the gut microbiota-gut-brain axis in regulating neurodegenerative diseases and cognitive impairment, including 24 keywords such as gut microbiota, metabolism, insulin-resistance, Parkinson’s disease, and glucagon-like peptide-1. Green Cluster 2 emphasized the neuroprotective effects of fatty acid dietary supplements against aging and type 2 diabetes, covering 22 keywords including inflammation, oxidative stress, aging, supplementation, mechanism, and neuroprotection. Blue Cluster 3 concentrated on the molecular mechanisms of SCFAs regulating cognitive function via the gut-brain axis, containing 21 keywords such as metabolites, memory function, gene-expression, receptor, and mice. Yellow Cluster 4 explored the associative mechanisms and diagnostic biomarkers linking the microbiota-gut-brain axis with neuropsychiatric disorders, involving 21 keywords including anxiety, schizophrenia, depression, association, and biomarkers. Purple Cluster 5 focused on the mechanisms of dietary interventions targeting the gut-brain axis in ischemic stroke, covering 19 keywords such as Mediterranean diet, dietary fiber, prebiotics, ischemic stroke, and gut-brain axis. Light Blue Cluster 6 centered on the mechanisms of SCFAs and exercise in targeting neuroinflammation to ameliorate cognitive decline in Alzheimer’s disease, including 16 keywords such as Alzheimer’s disease, neuroinflammation, exercise, blood-brain barrier, and butyrate.

**TABLE 9:** Top 10 keywords related to SCFAs and cognitive impairment.

WoSCC		Scopus	
Keyword	Count	Keyword	Count
Gut microbiota	236	Gut microbiota	417
Alzheimer’s disease	130	Alzheimer’s disease	202
microbiota	97	Nervous system inflammation	169
Neuroinflammation	84	Pathophysiology	169
Inflammation	81	Review	157
Gut-brain axis	65	Dysbiosis	154
Brain	61	Brain-gut axis	141
Mouse	61	Inflammation	139
Dementia	48	Microbiota	125
Oxidative stress	43	Probiotics	116

A total of 6268 keywords were identified from the Scopus database via VOSviewer. The top 10 keywords with occurrence > 183 times are listed in Table 9, reflecting the main research priorities of this field. Among them, “gut microbiota” showed the highest frequency (417 times), followed by “Alzheimer’s disease” (202 times), “nervous system inflammation” (169 times), “pathophysiology” (169 times), and “review” (157 times). Then, based on the threshold of keyword occurrence ≥ 20, 193 keywords were selected to draw a keyword clustering map (Figure 8B), and four distinct color-coded clusters were identified. Red Cluster 1 focused on the mechanisms and intervention strategies of SCFAs in regulating neuroinflammation and improving Alzheimer’s disease-related cognitive impairment via the gut-brain axis, including 75 keywords such as gut microbiota, Alzheimer’s disease, gut-brain axis, nervous system inflammation, prebiotic agent, and diet therapy. Green Cluster 2 concentrated on the neuroprotective and cognitive-improving effects of SCFAs, covering 67 keywords including animal experiment, Morris water maze test, antiinflammatory activity, neuroprotective agents, and antioxidant activity. Blue Cluster 3 explored biomarker identification and clinical characteristics of the gut microbiota-SCFA axis in aging-related cognitive impairment, including 37 keywords such as aging, middle aged, biological marker, priority journal, and bacteroides. Yellow Cluster 4 analyzed the mechanisms by which dietary fiber regulates cognitive impairment associated with neurodegenerative diseases through the gut microbiota-SCFA axis, including 12 keywords such as neurodegenerative diseases, dietary fiber, bacteria, metabolites, and transgenic mouse. These clusters collectively indicate that the interdisciplinary research on SCFAs and cognitive impairment holds diverse core values in basic medicine and clinical translation. It serves as a core component of basic research for elucidating the molecular mechanisms of the gut microbiota-SCFA-brain axis in regulating the pathological progression of cognitive impairment, neuroprotective pharmacological activities, and screening intervention targets. It also provides support for clinical translation, including the identification of early non-invasive biomarkers, analysis of clinical characteristics, precise dietary interventions, and development of prebiotic preparations for aging-related cognitive impairment.

**FIGURE 9:**
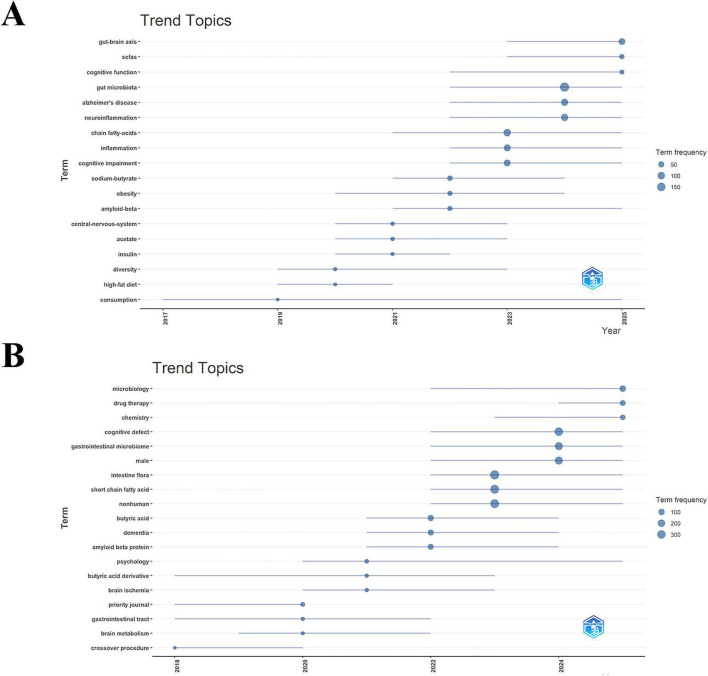
Trend topics on SCFAs and cognitive impairment. **(A)** Trend topics based on WoSCC. **(B)** Trend topics based on Scopus.

To explore the evolutionary trends of research themes in this field, we used the bibliometrix package in R software to generate a thematic trend map (Figure 9). The thematic trend map facilitates tracking the temporal development of specific research themes within a field, allowing us to monitor the evolution of research hotspots over time and gain deeper insights into its developmental process. Research themes have gradually evolved from exploring the basic associations between dietary behavior, gut microbiota diversity, and host metabolism, to dissecting the central nervous system regulation and pathological mechanisms of cognitive impairment mediated by short-chain fatty acids, and finally to translational research and clinical application exploration of targeted interventions on the gut microbiota-gut-brain axis for cognitive impairment in neurodegenerative diseases such as Alzheimer’s disease. Early studies from 2017 to 2020 focused on the associations among dietary intake, basic characteristics of gut microbiota, host metabolism, and the central nervous system, with keywords including “consumption,” “diversity,” “high-fat diet,” “acetate,” “central-nervous-system,” “insulin,” and “obesity.” Mid-term studies from 2021 to 2022 centered on the neurobiological effects of short-chain fatty acids, elucidation of pathological mechanisms related to cognitive impairment, and mining of intervention targets, with relevant terms including “sodium-butyrate,” “amyloid-beta,” “chain fatty-acids,” “inflammation,” and “cognitive impairment.” Since 2023, research has centered on “gut microbiota,” “Alzheimer’s disease,” “neuroinflammation,” “gut-brain axis,” “scfas,” and “cognitive function”, emphasizing the mechanisms of the gut microbiota-gut-brain axis-mediated SCFA regulatory pathways in the development of cognitive impairment in neurodegenerative diseases such as Alzheimer’s disease, as well as targeted intervention studies for cognitive protection. Emerging high-frequency terms such as “gut-brain axis,” “scfas,” and “cognitive function” further indicate that research is advancing toward precise targeted regulation of the gut-brain axis, early intervention of cognitive impairment in neurodegenerative diseases, and clinical translation.

Results from the Scopus database (Figure 9B) show that research themes have gradually progressed from exploring basic organ associations and research methods related to SCFAs, to dissecting the core components of SCFAs and pathological mechanisms of cognitive impairment, and finally to translational research and clinical intervention exploring the regulation of cognitive deficits by the gut microbiota-SCFA axis. Early studies from 2018 to 2020 focused on basic gut-brain associations, research design, and derivative components related to SCFAs, with keywords including “crossover procedure,” “priority journal,” “gastrointestinal tract,” “brain metabolism,” and “butyric acid derivative.” Mid-term studies from 2021 to 2022 concentrated on core components of SCFAs and the core pathology and phenotypic analysis of cognitive impairment, with relevant terms including “butyric acid,” “dementia,” and “amyloid beta protein.” Since 2023, research has centered on “intestine flora,” “short chain fatty acid,” “cognitive defect,” and “gastrointestinal microbiome,” emphasizing the regulatory role of the gut microbiota-SCFA axis in the development of cognitive deficits and exploration of targeted interventions. Emerging terms such as “drug therapy,” “microbiology,” and “chemistry” further indicate a shift toward precise targeted interventions of the gut microbiota-SCFA axis and multidisciplinary integrated clinical translation.

In summary, the results from both databases demonstrate that research on the regulation of cognitive function and neurodegenerative diseases by gut microbiota and its metabolite SCFAs via the gut-brain axis has evolved from early-stage exploration of basic associations among dietary behavior, gut microbiota diversity, host metabolism, and the central nervous system, to elucidation of the neurobiological effects of SCFAs, pathological mechanisms of cognitive impairment and neurodegeneration, and identification of intervention targets. Currently, research is progressively advancing toward precise targeted regulation of the gut microbiota-gut-brain axis, early intervention of cognitive impairment in neurodegenerative diseases such as Alzheimer’s disease, in-depth mechanistic research, and clinical translational applications.

### 3.6 Integrated analysis of research hotspots

Combining citation burst detection, keyword clustering, and thematic evolution analysis, this study identified three major research hotspots: (1) The mechanisms by which SCFAs regulate cognitive impairment through the microbiota-gut-brain axis. (2) Clinical characteristics and biomarkers of gut microbiota-SCFAs axis disturbance in Alzheimer’s disease and its prodromal stage, mild cognitive impairment. (3) Intervention strategies targeting the gut microbiota-SCFAs axis to ameliorate cognitive impairment, including clinical application explorations such as dietary interventions (modified ketogenic diet, intermittent fasting, Mediterranean diet), prebiotic/polysaccharide/fatty acid supplementation, fecal microbiota transplantation, and exercise rehabilitation.

## 4 Discussion

### 4.1 Basic information

This study constructed an integrated dataset consisting of 425 articles retrieved from WoSCC and 514 articles from Scopus published between 2009 and 2025. The results revealed a steep annual growth in publications investigating SCFAs and cognitive impairment over this period, reflecting sustained rising academic interest in this interdisciplinary field. In-depth analysis identified two core drivers fueling the rapid expansion of this research area. First, governments and authoritative research funding bodies worldwide have steadily increased financial investment, launching a series of special research programs and major funded projects focused on the gut microbiome, its metabolites, and cognitive impairment. Representative examples include China’s National Key Research and Development Program entitled Mechanism Research on Gut Microbiome Remodeling via Fecal Microbiota Transplantation, and the National Natural Science Foundation of China project Effects and Mechanisms of Intestinal Prevotella in Vascular Cognitive Impairment. These funded projects have supported extensive basic and clinical research on gut–brain axis regulatory mechanisms, the roles of gut microbial metabolites in the initiation and progression of cognitive impairment, and microecological intervention targets, delivering critical financial backing and policy guidance for the advancement of this field. China stands as the leading contributor with a substantially larger publication output than any other country. This dominant position can be attributed to its large patient population, abundant clinical research resources, national policy support, consistent research funding, mature interdisciplinary collaboration frameworks, and increasingly sophisticated clinical translation systems. At the institutional level, Wenzhou Medical University (China) and the University of California (USA) are the primary publishing entities and serve as central hubs within international collaboration networks. Nevertheless, while Chinese institutions produce the highest volume of publications, they have yet to establish extensive international collaborative partnerships, indicating substantial room for expanding and deepening global joint research on SCFAs and cognitive impairment. In terms of journal distribution, the 425 WoSCC articles span 372 academic journals, whereas the 514 Scopus articles are distributed across 241 journals. The core publication platforms include International Journal of Molecular Sciences and Nutrients, both classified as JCR Q1 journals, which demonstrates the generally high academic quality of research outputs in this domain. Further analysis of publication volume and citation metrics shows that International Journal of Molecular Sciences accommodates the largest number of relevant articles included in this study, while Nutrients ranks first in total citations, firmly establishing both journals as core academic platforms for SCFA and cognitive impairment research. Notably, several top high-impact journals in this field exhibit a distinct pattern of “low publication proportion but high single-article citation counts.” This phenomenon arises from their rigorous peer-review standards and strict acceptance thresholds. Studies published in these top journals concentrate on cutting-edge interdisciplinary topics with strong theoretical novelty and clinical implications. Even though they do not constitute the primary source of literature in our dataset, their publications provide foundational theoretical support and receive widespread attention and citations from global researchers. Researchers in this field have formed extensive, tight-knit academic collaboration networks. Scholars including Zhang, Xin, Liu, Jiaming, Liu, Xuebo, Liu, Zhigang, and Sun, Jing possess prominent academic influence, among whom Zhang, Xin represents the core author dedicated to SCFA and cognitive impairment research.

### 4.2 Research hotspots and trends

By integrating literature clustering, keyword frequency analysis, co-occurrence mapping, and thematic evolution analysis, this study identified three major research hotspots in the field of SCFAs and cognitive impairment.

#### 4.2.1 Mechanisms underlying SCFAs-mediated regulation of cognitive impairment via the microbiota-gut-brain axis

SCFAs act as key messengers that “transmit” signals from the gut microbiota to the brain. They maintain brain health and modulate cognitive function by regulating multiple molecular signaling pathways. First, animal studies have demonstrated that SCFAs (e.g., butyrate) suppress the nuclear factor-kappa B (NF-κB) signaling pathway in mice, reduce the activation of astrocytes and microglia, decrease the release of pro-inflammatory cytokines (e.g., IL-6, TNF-α), and alleviate neuroinflammatory responses (30, 31). Neuroinflammation is an important pathological mechanism underlying cognitive impairment (e.g., AD, dementia) (32), and the anti-inflammatory effects of SCFAs help protect neurons from inflammatory damage and preserve normal cognitive function (21). Second, SCFAs upregulate the expression of tight junction proteins ZO-1 and Occludin, enhancing the integrity of the blood-brain barrier (BBB) (33), which helps reduce the entry of harmful substances (e.g., lipopolysaccharide) into the brain and prevents neuronal damage (34). Impaired BBB function is an early marker of cognitive decline, and SCFAs provide a protective environment for the brain by maintaining BBB stability (35). Third, *in vivo* and *in vitro* studies have shown that SCFAs can not only reduce the deposition of pathological amyloid-β protein, but also upregulate the expression of brain-derived neurotrophic factor (BDNF), promote synaptogenesis and neuroplasticity, strengthen interneuronal connections, and ultimately enhance learning and memory capacities in the brain (36). Finally, SCFAs can influence the synthesis and release of neurotransmitters, such as promoting the production of γ-aminobutyric acid (GABA), 5-hydroxytryptamine (5-HT), and other neurotransmitters (37, 38). These neurotransmitters are essential for cognitive functions including learning, memory, and emotional regulation, and SCFAs may improve cognitive performance by balancing neurotransmitter levels (39).

In summary, SCFAs coordinately form a multidimensional regulatory network involving anti-inflammatory signaling, maintenance of BBB integrity, reduction of pathological protein burden, promotion of neurotrophic factor expression, and modulation of neurotransmitter synthesis and release, ultimately mediating the cognitive protective effects of SCFAs. These mechanisms provide a strong rationale for the clinical application of endogenous or exogenous SCFAs interventions (diet, exercise, probiotics) in cognitive impairment and related disorders.

#### 4.2.2 Clinical characteristics and biomarkers of gut microbiota-SCFAs axis dysregulation in AD and its prodromal stage of mild cognitive impairment

AD is an irreversible neurodegenerative disease, and mild cognitive impairment (MCI), as its prodromal stage, represents a critical window for early intervention (40). Recent studies have confirmed that gut microbiota-SCFAs axis dysregulation participates in the pathogenesis of AD and MCI via the gut-brain axis (41), and research into its clinical characteristics and biomarkers offers new directions for early diagnosis and intervention.

Clinical features of gut microbiota-SCFAs axis dysregulation exhibit stage-specific differences. Significant microbial imbalance is already present at the MCI stage, with decreased α-diversity, reduced abundance of SCFAs-producing bacteria such as Faecalibacterium and Roseburia, and an increased proportion of Bacteroidota, with these changes preceding abnormalities in brain pathological indicators (42, 43). Upon progression to AD, microbial dysregulation is further aggravated, with enrichment of Proteobacteria and Actinobacteriota, continuous reduction of beneficial bacteria including Bifidobacterium and Lactobacillus, and a positive correlation between pro-inflammatory microbiota abundance and cerebral amyloid-beta deposition (44, 45). As key microbial metabolites, SCFAs levels are significantly reduced at the MCI stage, especially acetate, butyrate, and propionate, and correlate positively with cognitive function scores (46); in females, propionate levels are also associated with tau phosphorylation accumulation and the rate of cognitive decline (47).

Relevant biomarkers show promising clinical application potential. For microbial markers, Faecalibacterium is decreased in MCI patients (48), while Akkermansia and Escherichia are enriched in AD patients, whose dynamic changes can reflect disease progression (49). Among SCFAs-related biomarkers, plasma or fecal acetate levels have been found to trend downward in MCI patients, with changes positively correlated with cognitive test scores (e.g., Montreal Cognitive Assessment scores), indicating diagnostic potential (50). Butyrate levels correlate with multiple dimensions of cognitive function (e.g., memory, executive function), and its changes may reflect disease progression (51). Moreover, SCFA levels are associated with pathological markers in cerebrospinal fluid such as Aβ42/Aβ40 and tau protein (52). In addition, changes in the abundance of bacteria harboring propionate and butyrate synthesis genes may serve as potential markers for disease risk assessment.

In summary, gut microbiota-SCFAs axis dysregulation is a key feature of AD and MCI, and the associated microbial and SCFAs biomarkers provide novel targets for early disease screening and progression monitoring. Future standardized longitudinal studies are needed to clarify the causal relationship between axis dysregulation and disease and to promote clinical translation.

#### 4.2.3 Intervention strategies targeting the gut microbiota-SCFAs axis to ameliorate cognitive impairment

In recent years, the mechanisms by which the gut microbiota-SCFAs axis regulates central nervous system function through the gut-brain axis have been widely confirmed. As key metabolites of the gut microbiota, SCFAs can improve cognitive function by inhibiting neuroinflammation, protecting the blood-brain barrier, and regulating neurotransmitter secretion (53). Currently, intervention strategies targeting this axis have gradually entered clinical exploration, covering dietary intervention, nutritional supplementation, fecal microbiota transplantation, exercise rehabilitation, and other fields, providing new approaches for non-pharmacological intervention of cognitive impairment (54).

Dietary intervention is a core strategy for regulating the gut microbiota-SCFAs axis, as it modulates gut microbiota composition and metabolism to improve cognitive function. Modified ketogenic diets optimize the ratio of fat, protein, and carbohydrates with the addition of fermentable fiber and ω-3 polyunsaturated fatty acids, significantly enriching SCFAs-producing bacteria such as Faecalibacterium and Bifidobacterium, restoring acetate and butyrate levels and the butyrate/propionate ratio, and avoiding metabolic side effects of classic ketogenic diets (55, 56). Clinical studies have shown that such diets can improve cognitive scores in patients with cognitive impairment and reduce excessive deposition of pathological proteins in the brain (41). Intermittent fasting (e.g., 16:8 pattern) regulates gut microbiota diversity, promotes SCFA synthesis, improves intestinal barrier function, and reduces neuroinflammation (57), making it particularly suitable for cognitively impaired individuals with comorbid metabolic abnormalities; long-term intervention can delay cognitive decline (58). The Mediterranean diet emphasizes high dietary fiber, high-quality fats, and fresh fruits and vegetables. A 3-year follow-up study confirmed that individuals with high adherence had a significantly lower risk of cognitive decline, with long-term intake of legumes, fish, and vegetables effectively protecting cognitive function, especially in women over 65 years old (59); its mechanism is closely related to increased beneficial bacteria abundance and elevated SCFAs levels (60). In conclusion, modified ketogenic diets, intermittent fasting, and the Mediterranean diet can all improve brain cognitive function by targeting the gut microbiota-SCFAs axis. Although large-scale randomized trials are needed to confirm long-term adherence and safety, current evidence supports these dietary patterns as components of comprehensive management for cognitive impairment.

Supplementation with prebiotics, polysaccharides, and fatty acids is an important approach for precise regulation of the gut microbiota-SCFAs axis, with advantages of high safety and strong specificity. Prebiotics (e.g., fructooligosaccharides, inulin) can selectively promote the proliferation of SCFAs-producing bacteria and inhibit harmful bacteria (61, 62). One study showed that 12 weeks of combined Bifidobacterium and inulin intervention significantly improved MoCA scores and gut microbiota diversity in cognitively impaired patients (63). Plant polysaccharides (e.g., Lycium barbarum polysaccharide, Tremella polysaccharide) regulate gut microbiota balance, promote SCFAs synthesis, and protect neurons through antioxidant and anti-inflammatory effects, providing natural and safe options for cognitive impairment intervention (64, 65). Notably, the bioactive peptide RW-9 derived from oat protein exerts beneficial effects on neuroprotection and the prevention of neurodegenerative diseases (66). It can modulate gut microbiota composition and the corresponding production of SCFAs in AD mice, inhibit cerebral oxidative stress and inflammatory responses, and consequently ameliorate cognitive deficits (67, 68).

Exercise training regulates the gut microbiota-SCFAs axis through multiple pathways to assist in improving cognitive function and can act synergistically with other intervention strategies. Moderate-intensity aerobic exercise increases gut microbiota diversity, promotes SCFAs production, improves cerebral blood circulation, reduces amyloid-beta deposition, and lowers oxidative stress (69, 70); resistance training modulates intestinal metabolism and enhances the neuroprotective effects of SCFAs (71). Clinical evidence confirms that regular exercise significantly improves memory and executive function in cognitively impaired patients and delays disease progression. Furthermore, the combined intervention of oat bran and exercise can more effectively boost the production of SCFAs by specific probiotic genera, alleviate systemic inflammation and regulate energy metabolism, thereby exerting potential protective effects on cognitive function (72). This indicates that combined dietary and exercise intervention represents a promising research direction for future investigations.

In summary, various intervention strategies targeting the gut microbiota-SCFA axis show research potential for the early intervention of cognitive impairment. Among them, dietary interventions and nutritional supplements are suitable for widespread clinical application due to their high safety and easy implementation, while personalized exercise rehabilitation programs should be formulated according to individual patient conditions. Standardized clinical trials are required in the future to determine the optimal dosage, intervention duration and applicable populations of each intervention strategy, address key challenges in clinical translation, facilitate their standardized clinical practice, and provide novel clinical approaches for the early intervention and treatment of cognitive impairment.

### 4.3 Differences between the WoSCC and Scopus databases and their impacts on the identification of research hotspots

This study incorporated both the WoSCC and Scopus databases for bibliometric analysis, systematically depicting the overall knowledge structure and developmental trajectory of SCFA-cognitive impairment research from complementary perspectives. Notably, this study did not assume equivalence between the two databases in publication volume, disciplinary coverage, or research types; instead, it fully accounted for their longstanding structural differences and intentionally adopted a dual-database strategy to enhance the comprehensiveness and robustness of the findings. In this analysis, Scopus indexed more relevant publications than WoSCC (425 vs. 514 articles). This difference mainly stems from systematic variations in journal selection criteria and disciplinary coverage: WoSCC prioritizes journal academic impact and stability, favoring high-impact journals with solid theoretical foundations and rigorous methodologies. In contrast, Scopus provides broader coverage of clinical medicine, public health, and applied research, with greater inclusivity for publication types such as clinical studies, observational studies, and case reports. Since SCFA-cognitive impairment research is inherently clinically oriented, and clinical interventions are often first explored via small-sample clinical studies or case reports, it is expected that Scopus would capture more relevant literature. This database-specific characteristic is also reflected in keyword analysis: compared with WoSCC, the Scopus dataset contains more high-frequency keywords related to clinical practice, case observation, and study types (e.g., clinical articles, case reports), with more refined terminology for clinical research, including human, aged, female, adult, and clinical outcome (Figure 8 and Table 9). This pattern does not indicate a shift in research themes but highlights Scopus’s strength in capturing real-world clinical research and early exploratory studies.

### 4.4 Limitations

This study systematically analyzes research concerning SCFAs and cognitive impairment based on two major databases, namely WoSCC and Scopus, and comprehensively sorts out publication trends, research hotspots and thematic evolution trajectories in this field. Nevertheless, several limitations of this work should be acknowledged. First, only WoSCC and Scopus were selected for analysis, while relevant studies indexed in other databases such as PubMed and Embase were not covered, introducing certain inclusion bias. Second, only English publications were retrieved, and studies written in other languages were excluded, which impairs the global representativeness of the results to some extent. Third, the self-developed search strategy may fail to capture a small number of articles adopting non-standard or niche terminologies, leading to potential incomplete retrieval bias. Fourth, inherent patterns of academic citation tend to overestimate the academic weight of classic reviews and studies with positive findings, making it impossible to fully reflect the genuine contributions of all types of research within the field. In addition, the retrieval window was set from 2009 to 2025. Some articles published in late 2025 may not have been fully indexed by the databases, which could generate minor deviations in the statistics of annual publication output. Finally, restricted by the data export format of Scopus, co-citation analysis was conducted solely using WoSCC datasets, which somewhat limits the scope of knowledge mapping. Despite the above limitations, the dual-database cross-verification design ensures the fundamental reliability and robustness of our core findings. The results can still offer valuable references for understanding the overall research landscape and emerging frontiers in the field of SCFAs and cognitive impairment.

## 5 Conclusion

Through cross-verification of datasets retrieved from two independent databases, this study systematically identifies the core research hotspots and emerging frontiers regarding SCFAs and cognitive impairment. The major developmental trajectory and research trends of this field are summarized as follows:

a. Research related to SCFAs and cognitive impairment has received extensive global attention. Countries such as China and the United States represent the core research forces in this field, with high research activity and close international cooperation. With the continuous advancement of relevant studies, international collaboration will be further strengthened in the future.
b. In this field, *International Journal of Molecular Sciences* is the journal with the largest number of publications, and *Nutrients* is the most frequently cited journal, fully reflecting their representative status as core academic platforms in this discipline.
c. Co-authorship analysis of publications on SCFAs and cognitive impairment reveals extensive and close collaboration among researchers. Zhang, Xin and Liu, Jiaming, among others, are representative core authors in this field.

d. The mechanisms by which SCFAs regulate cognitive impairment through the microbiota–gut–brain axis have become a major research hotspot in this field.
e. The clinical characteristics and biomarkers of gut microbiota–SCFAs axis dysregulation in AD and its prodromal stage of mild cognitive impairment represent a key research hotspot and developmental trend in this field.
f. Intervention strategies targeting the gut microbiota–SCFAs axis to improve cognitive impairment have emerged as an important research trend in this field.

In summary, this study provides valuable references for analyzing research trends and hotspots in the field of SCFAs and cognitive impairment. The above findings help researchers quickly grasp the current research status and explore future directions. This study also identifies the limitations of current research and potential key directions, providing guidance for researchers to deepen relevant studies and develop innovative research ideas.

## Data Availability

The original contributions presented in the study are included in the article/supplementary material, further inquiries can be directed to the corresponding authors.

## References

[1] Han X Pan C Cai Z Zhang A Zhong N Pu L et al. The association between sensory function changes, metabolic and inflammatory biomarkers, and cognitive impairment: two prospective cohort studies. *J Gerontology A Biol Sci Med Sci.* (2025) 80:glaf160. 10.1093/gerona/glaf160 40690389

[2] Reineke LC Zhu PJ Dalwadi U Dooling SW Liu Y Wang I et al. Harnessing viral strategies to reverse cognitive dysfunction through the integrated stress response. *Science (New York, N.Y.).* (2026) 392:eaea8782. 10.1126/science.aea8782 41926581

[3] Ornish D Madison C Kivipelto M Kemp C McCulloch CE Galasko D et al. Effects of intensive lifestyle changes on the progression of mild cognitive impairment or early dementia due to Alzheimer’s disease: a randomized, controlled clinical trial. *Alzheimers Res Ther.* (2024) 16:122. 10.1186/s13195-024-01482-z 38849944 PMC11157928

[4] Lorenz SM Wahida A Bostock MJ Seibt T Santos Dias Mourão A Levkina A et al. A fin-loop-like structure in gpx4 underlies neuroprotection from ferroptosis. *Cell.* (2026) 189:287–306. 10.1016/j.cell.2025.11.014 41349546

[5] Grünewald B Wickel J Hahn N Rahmati V Rupp H Chung H et al. Targeted rescue of synaptic plasticity improves cognitive decline in sepsis-associated encephalopathy. *Mol Ther.* (2024) 32:2113–29. 10.1016/j.ymthe.2024.05.001 38788710 PMC11286813

[6] Riffo-Lepe N González-Sanmiguel J Armijo-Weingart L Saavedra-Sieyes P Hernandez D Ramos G et al. Synaptic and synchronic impairments in subcortical brain regions associated with early non-cognitive dysfunction in Alzheimer’s disease. *Neural Regen Res.* (2026) 21:248–64. 10.4103/NRR.NRR-D-24-01052 39885666 PMC12094569

[7] Udeochu JC Amin S Huang Y Fan L Torres ERS Carling GK et al. Tau activation of microglial CGAS-IFN reduces MEF2C-mediated cognitive resilience. *Nat Neurosci.* (2023) 26:737–50. 10.1038/s41593-023-01315-6 37095396 PMC10166855

[8] Akhtar M Rafique H Alam Y Khalid MZ Zhang J Alsulami T et al. Pectin (RG-1)-like polysaccharides isolated from Gastrodiae rhizoma via fractional ethanol precipitation: potent inhibitors of pro inflammatory enzyme modulation targeting iNOS and COX-2. *Int J Biol Macromol.* (2025) 322:146784. 10.1016/j.ijbiomac.2025.146784 40812638

[9] Ali Z Rafique H Tahir RA Saeed T Rasheed MA Khan I et al. Clinical and computational exploration of red date fruit vinegar: synergistic effects on cardiovascular and type 2 diabetes pathways. *Front Nutr.* (2025) 12:1557733. 10.3389/fnut.2025.1557733 40727700 PMC12302754

[10] Duan H Zhou D Xu N Yang T Wu Q Wang Z et al. Association of unhealthy lifestyle and genetic risk factors with mild cognitive impairment in Chinese older adults. *JAMA Netw Open.* (2023) 6:e2324031. 10.1001/jamanetworkopen.2023.24031 37462970 PMC10354670

[11] Lee J Han K Kim J Lim J Cheon DY Lee M. Association between metabolic syndrome and young-onset dementia: a nationwide population-based study. *Neurology.* (2025) 104:e213599. 10.1212/WNL.0000000000213599 40267374

[12] Livingston G Huntley J Liu KY Costafreda SG Selbæk G Alladi S et al. Dementia prevention, intervention, and care: 2024 report of the lancet standing commission. *Lancet (Lond, Engl).* (2024) 404:572–628. 10.1016/S0140-6736(24)01296-0 39096926

[13] Frisoni GB Hansson O Nichols E Garibotto V Schindler SE van der Flier WM et al. New landscape of the diagnosis of Alzheimer’s disease. *Lancet (Lond, Engl).* (2025) 406:1389–407. 10.1016/S0140-6736(25)01294-2 40997838

[14] Shen L Dewan P Ferreira JP Cunningham JW Jhund PS Anand IS et al. Clinical correlates and prognostic impact of cognitive dysfunction in patients with heart failure and preserved ejection fraction: insights from PARAGON-HF. *Circulation.* (2024) 150:1913–27. 10.1161/CIRCULATIONAHA.124.070553 39429145

[15] Jian Y Jia S Shi S Shi Z Zhao Y. A nomogram to predict the risk of cognitive impairment in patients with depressive disorder. *Res Nurs Health.* (2024) 47:302–11. 10.1002/nur.22364 38149849

[16] Chen S Cao Z Nandi A Counts N Jiao L Prettner K et al. The global macroeconomic burden of Alzheimer’s disease and other dementias: estimates and projections for 152 countries or territories. *Lancet Glob Health.* (2024) 12:e1534–43. 10.1016/S2214-109X(24)00264-X 39151988

[17] Cox TO Devason AS de Araujo A Mason S Subramanian M Salvador AFM et al. Intestinal interoceptive dysfunction drives age-associated cognitive decline. *Nature.* (2026) 652:442–50. 10.1038/s41586-026-10191-6 41813891 PMC13061634

[18] Mukhopadhya I Louis P. Gut microbiota-derived short-chain fatty acids and their role in human health and disease. *Nat Rev Microbiol.* (2025) 23:635–51. 10.1038/s41579-025-01183-w 40360779

[19] You M Chen N Yang Y Cheng L He H Cai Y et al. The gut microbiota-brain axis in neurological disorders. *Medcomm (2020).* (2024) 5:e656. 10.1002/mco2.656 39036341 PMC11260174

[20] Zhang Y Jiang H Peng X Zhao Y Huang X Yuan K et al. Mulberry leaf improves type 2 diabetes in mice via gut microbiota-SCFAs-GPRs axis and AMPK signaling pathway. *Phytomedicine.* (2025) 145:156970. 10.1016/j.phymed.2025.156970 40527063

[21] Li Q Zhang-Yang Q Tan Y Wu X Zha X Shang Z et al. *Dendrobium huoshanense* stem polysaccharide protects against Alzheimer’s disease by modulating SCFAs/GLP-1 axis-mediated neuroinflammation suppression. *Int J Biol Macromol.* (2026) 353:151235. 10.1016/j.ijbiomac.2026.151235 41794249

[22] Wang J Xie J He F Wu W Xu K Ren Y et al. *Akkermansia muciniphila*-derived SCFAs improve the depression-like behaviors of mice by inhibiting neuroinflammation. *Pharmacol Res.* (2025) 220:107938. 10.1016/j.phrs.2025.107938 40886782

[23] Mann ER Lam YK Uhlig HH. Short-chain fatty acids: linking diet, the microbiome and immunity. *Nat Rev Immunol.* (2024) 24:577–95. 10.1038/s41577-024-01014-8 38565643

[24] Zang J Xiao L Shi Y Kou Y Ma K Zhang C et al. Advances in dietary modulation of the intestinal barrier: mechanistic, structural, and functional insights. *Compr Rev Food Sci Food Saf.* (2026) 25:e70383. 10.1111/1541-4337.70383 41511109

[25] Li S Heng X Guo L Lessing DJ Chu W. SCFAs improve disease resistance via modulate gut microbiota, enhance immune response and increase antioxidative capacity in the host. *Fish Shellfish Immunol.* (2022) 120:560–8. 10.1016/j.fsi.2021.12.035 34958920

[26] Hays KE Pfaffinger JM Ryznar R. The interplay between gut microbiota, short-chain fatty acids, and implications for host health and disease. *Gut Microbes.* (2024) 16:2393270. 10.1080/19490976.2024.2393270 39284033 PMC11407412

[27] Brinck JE Sinha AK Laursen MF Dragsted LO Raes J Uribe RV et al. Intestinal pH: a major driver of human gut microbiota composition and metabolism. *Nat Rev Gastroenterol Hepatol.* (2025) 22:639–56. 10.1038/s41575-025-01092-6 40603778

[28] Pu D Jin Y Wang L Wang R Li L Song Y et al. Combined supplementation of short-chain fatty acids reduces hyperphosphorylation of Tau at T181,T231 and S396 sites and improves cognitive impairment in a chemically induced AD mouse model via regulation of HDAC and KEAP1. *Neurochem Int.* (2025) 189:106034. 10.1016/j.neuint.2025.106034 40812734

[29] Xi X Hu S Hou W Qin Y Wu C Luo L et al. A bibliometric analysis of the mediterranean diet in metabolic syndrome (2015-2025). *Front Nutr.* (2026) 13:1765074. 10.3389/fnut.2026.1765074 41626613 PMC12855129

[30] Wang X Wang S Zheng B Guo Z. Lotus seed starch-chlorogenic acid complexes alleviated chronic colitis and linked cognitive dysfunction via gut microbiota regulation: gut-brain-axis interaction. *Carbohydr Polym.* (2025) 368:124158. 10.1016/j.carbpol.2025.124158 40947251

[31] Wei H Yu C Zhang C Ren Y Guo L Wang T et al. Butyrate ameliorates chronic alcoholic central nervous damage by suppressing microglia-mediated neuroinflammation and modulating the microbiome-gut-brain axis. *Biomed Pharmacother.* (2023) 160:114308. 10.1016/j.biopha.2023.114308 36709599

[32] Guo S Jin H Sun H Huang S Chen Y Chang Y et al. TDP-43 pathology triggers neuroinflammation and cognitive impairment by inducing microglial necroptosis. *Embo Mol Med.* (2026) 18:1318–41. 10.1038/s44321-026-00394-9 41807703 PMC13083925

[33] Song Y Deng Q Li J Chen R Li D Wang S et al. Structure-activity relationships and mechanisms of natural polysaccharides in modulating neurological disorders via the microbiota-gut-brain axis. *Carbohydr Polym.* (2025) 367:123960. 10.1016/j.carbpol.2025.123960 40817517

[34] Liu S Wang M Meng X Pan J Fang J Cheng W et al. Flavonoids from Shiliangcha (*Chimonanthus salicifolius*) alleviate brain aging in d-galactose-induced senescent mice through gut microbiota. *J Agric Food Chem.* (2025) 73:16890–905. 10.1021/acs.jafc.5c00835 40388484

[35] Friedman A Prager O Serlin Y Kaufer D. Dynamic modulation of the blood-brain barrier in the healthy brain. *Nat Rev Neurosci.* (2025) 26:749–64. 10.1038/s41583-025-00976-5 41094205

[36] Shi M Yang J Liu Y Zhao H Li M Yang D et al. Huanglian wendan decoction improves insomnia in rats by regulating BDNF/TrkB signaling pathway through gut microbiota-mediated SCFAs and affecting microglia polarization. *Mol Neurobiol.* (2025) 62:1047–66. 10.1007/s12035-024-04330-1 38954253

[37] Shen H Zhang C Zhang Q Lv Q Liu H Yuan H et al. Gut microbiota modulates depressive-like behaviors induced by chronic ethanol exposure through short-chain fatty acids. *J Neuroinflammation.* (2024) 21:290. 10.1186/s12974-024-03282-6 39508236 PMC11539449

[38] Gao F Su J Li J Gan C Jin X Zhou H et al. Study on the effects of the atractylodes macrocephala koidz-raphanus sativus l herb pair in alleviating senile constipation via the gut microbiota-SCFAs-5-HT axis. *Phytomedicine.* (2025) 145:156977. 10.1016/j.phymed.2025.156977 40554896

[39] Dicks LMT. Our mental health is determined by an intrinsic interplay between the central nervous system, enteric nerves, and gut microbiota. *Int J Mol Sci.* (2023) 25:38. 10.3390/ijms25010038 38203207 PMC10778721

[40] Li L Jia M Yang C Zhao Y Hu J Zhao Y et al. Gut microbial-derived indole-3-propionate improves cognitive function in Alzheimer’s disease. *Sci Adv.* (2025) 11:eadw8410. 10.1126/sciadv.adw8410 41313780 PMC12662223

[41] Du L Chen J Yan J Xie H Wang L Wang R et al. Lingguizhugan decoction ameliorates cognitive impairment in ad-like mice by influencing the microbiome-gut-brain axis mediated by SCFAs. *Phytomedicine.* (2024) 133:155942. 10.1016/j.phymed.2024.155942 39173279

[42] Chen G Zhou X Zhu Y Shi W Kong L. Gut microbiome characteristics in subjective cognitive decline, mild cognitive impairment and Alzheimer’s disease: a systematic review and meta-analysis. *Eur J Neurol.* (2023) 30:3568–80. 10.1111/ene.15961 37399128

[43] Pan Q Li Y Guo K Xue M Gan Y Wang K et al. Elderly patients with mild cognitive impairment exhibit altered gut microbiota profiles. *J Immunol Res.* (2021) 2021:5578958. 10.1155/2021/5578958 34869782 PMC8635943

[44] Jia L Ke Y Zhao S Liu J Luo X Cao J et al. Metagenomic analysis characterizes stage-specific gut microbiota in Alzheimer’s disease. *Mol Psychiatry.* (2025) 30:3951–62. 10.1038/s41380-025-02973-7 40164697

[45] Liu J Cao J Jia L Gan Z Zhao X Yang A et al. Impacts of host genetics on gut microbiome composition in Alzheimer’s disease. *Microbiome.* (2026) 14:115. 10.1186/s40168-026-02342-8 41782023 PMC13069797

[46] Li S Yang P Cai X He M He Y He F. Vitamin c supplementation mitigates mild cognitive impairment in mice subjected to d-galactose: insights into intestinal flora and derived SCFAs. *Eur J Pharmacol.* (2025) 1001:177787. 10.1016/j.ejphar.2025.177787 40449647

[47] Chandra S Popovic J Singhal NK Watkins EA Dodiya HB Weigle IQ et al. The gut microbiome controls reactive astrocytosis during Aβ amyloidosis via propionate-mediated regulation of IL-17. *J Clin Investigat.* (2025) 135:e180826. 10.1172/JCI180826 40359034 PMC12208551

[48] Ueda A Shinkai S Shiroma H Taniguchi Y Tsuchida S Kariya T et al. Identification of *Faecalibacterium prausnitzii* strains for gut microbiome-based intervention in Alzheimer’s-type dementia. *Cell Rep Med.* (2021) 2:100398. 10.1016/j.xcrm.2021.100398 34622235 PMC8484692

[49] Wang Z Wang C Yuan B Liu L Zhang H Zhu M et al. *Akkermansia muciniphila* and its metabolite propionic acid maintains neuronal mitochondrial division and autophagy homeostasis during Alzheimer’s disease pathologic process via GPR41 and GPR43. *Microbiome.* (2025) 13:16. 10.1186/s40168-024-02001-w 39833898 PMC11744907

[50] Zhao Y Song P Zhang H Chen X Han P Yu X et al. Alteration of plasma metabolic profile and physical performance combined with metabolites is more sensitive to early screening for mild cognitive impairment. *Front Aging Neurosci.* (2022) 14:951146. 10.3389/fnagi.2022.951146 35959293 PMC9360416

[51] Ge X Zheng M Hu M Fang X Geng D Liu S et al. Butyrate ameliorates quinolinic acid-induced cognitive decline in obesity models. *J Clin Investigat.* (2023) 133:e154612. 10.1172/JCI154612 36787221 PMC9927952

[52] Nagpal R Neth BJ Wang S Craft S Yadav H. Modified mediterranean-ketogenic diet modulates gut microbiome and short-chain fatty acids in association with Alzheimer’s disease markers in subjects with mild cognitive impairment. *Ebiomedicine.* (2019) 47:529–42. 10.1016/j.ebiom.2019.08.032 31477562 PMC6796564

[53] He Q Ji L Wang Y Zhang Y Wang H Wang J et al. Acetate enables metabolic fitness and cognitive performance during sleep disruption. *Cell Metab.* (2024) 36:1998–2014. 10.1016/j.cmet.2024.07.019 39163862

[54] Zhao Y Jia M Ding C Bao B Li H Ma J et al. Time-restricted feeding mitigates Alzheimer’s disease-associated cognitive impairments via a *B. pseudolongum*-propionic acid-FFAR3 axis. *Imeta.* (2025) 4:e70006. 10.1002/imt2.70006 40236783 PMC11995186

[55] Chen M He Y Dong X Liu H Yan Z Lu X et al. Ketogenic diet inhibits glioma progression by promoting gut microbiota-derived butyrate production. *Cancer Cell.* (2025) 43:2119–35. 10.1016/j.ccell.2025.09.002 41005305

[56] Wang S Bao Z Li Z Zhao M Wang X Liu F. The impact of very-low-calorie ketogenic diets on gut microbiota in individuals with obesity: a systematic review and meta-analysis. *Gut Microbes.* (2025) 17:2566305. 10.1080/19490976.2025.2566305 41054273 PMC12505515

[57] Ceperuelo-Mallafré V Rodríguez-Peña M Badia J Villanueva-Carmona T Cedó L Marsal-Beltran A et al. Dietary switch and intermittent fasting ameliorate the disrupted postprandial short-chain fatty acid response in diet-induced obese mice. *Ebiomedicine.* (2025) 117:105827. 10.1016/j.ebiom.2025.105827 40561776 PMC12240114

[58] Liu Z Dai X Zhang H Shi R Hui Y Jin X et al. Gut microbiota mediates intermittent-fasting alleviation of diabetes-induced cognitive impairment. *Nat Commun.* (2020) 11:855. 10.1038/s41467-020-14676-4 32071312 PMC7029019

[59] Ni J Hernández-Cacho A Nishi SK Babio N Belzer C Konstati P et al. Mediterranean diet, gut microbiota, and cognitive decline in older adults with obesity/overweight and metabolic syndrome: a prospective cohort study. *Bmc Med.* (2025) 23:669. 10.1186/s12916-025-04488-y 41327300 PMC12667113

[60] Liu Y Gu X Li Y Wang F Vyas CM Peng C et al. Interplay of genetic predisposition, plasma metabolome and mediterranean diet in dementia risk and cognitive function. *Nat Med.* (2025) 31:3790–800. 10.1038/s41591-025-03891-5 40855194 PMC12618253

[61] Zhao Z Zhao Y Dai S Ge Y Ye J Shi R et al. Fructo-oligosaccharides restore high-fat diet-disrupted diurnal fluctuations in cognitive function in mice via the gut-brain axis. *J Agric Food Chem.* (2025) 73:30331–44. 10.1021/acs.jafc.5c10321 41201833

[62] Sun Y Ho C Zhang Y Hong M Zhang X. Plant polysaccharides utilized by gut microbiota: new players in ameliorating cognitive impairment. *J Tradit Complement Med.* (2022) 13:128–34. 10.1016/j.jtcme.2022.01.003 36970456 PMC10037067

[63] Azuma N Mawatari T Saito Y Tsukamoto M Sampei M Iwama Y. Effect of continuous ingestion of bifidobacteria and dietary fiber on improvement in cognitive function: a randomized, double-blind, placebo-controlled trial. *Nutrients.* (2023) 15:4175. 10.3390/nu15194175 37836458 PMC10574581

[64] Tian X Dong W Zhou W Yan Y Lu L Mi J et al. The polysaccharides from the fruits of *Lycium barbarum* ameliorate high-fat and high-fructose diet-induced cognitive impairment via regulating blood glucose and mediating gut microbiota. *Int J Biol Macromol.* (2024) 258:129036. 10.1016/j.ijbiomac.2023.129036 38151081

[65] Lee Q Xue Z Luo Y Lin Y Lai M Xu H et al. Low molecular weight polysaccharide of *Tremella fuciformis* exhibits stronger antioxidant and immunomodulatory activities than high molecular weight polysaccharide. *Int J Biol Macromol.* (2024) 281:136097. 10.1016/j.ijbiomac.2024.136097 39353518

[66] Rafique H Hu X Ren T Dong R Aadil RM Zou L et al. Characterization and exploration of the neuroprotective potential of oat-protein-derived peptides in pc12 cells and scopolamine-treated zebrafish. *Nutrients.* (2023) 16:117. 10.3390/nu16010117 38201947 PMC10780882

[67] Rafique H Dong R Tianqi L Ma Z Hu X Khalid MZ et al. Oat peptide ameliorates cognitive impairment via mediating gut-brain axis in mice: a multi-omics approach. *J Agric Food Res.* (2025) 24:102394. 10.1016/j.jafr.2025.102394

[68] Dong R Peng K Shi L Niu Q Rafique H Liu Y et al. Oat bran prevents high-fat-diet induced muscular dysfunction, systemic inflammation and oxidative stress through reconstructing gut microbiome and circulating metabolome. *Food Res Int (Ottawa, Ont.).* (2023) 172:113127. 10.1016/j.foodres.2023.113127 37689892

[69] Cintado E Muela P Martín-Rodríguez L Alcaide I Tezanos P Vlckova K et al. Gut microbiota regulates exercise-induced hormetic modulation of cognitive function. *Ebiomedicine.* (2025) 119:105876. 10.1016/j.ebiom.2025.105876 40768832 PMC12789708

[70] Varghese S Rao S Khattak A Zamir F Chaari A. Physical exercise and the gut microbiome: a bidirectional relationship influencing health and performance. *Nutrients.* (2024) 16:3663. 10.3390/nu16213663 39519496 PMC11547208

[71] Reljic D Hermann HJ Dieterich W Neurath MF Zopf Y. Exercise improves gut microbial metabolites in an intensity-dependent manner: a pooled analysis of randomized controlled trials. *Gut Microbes.* (2025) 17:2579354. 10.1080/19490976.2025.2579354 41320950 PMC12674298

[72] Dong R Rafique H Niu Q Zeng X Messia MC Yuan L et al. Interaction of oat bran and exercise training improved exercise adaptability via alleviating oxidative stress and promoting energy homeostasis. *Food Funct.* (2024) 15:11508–24. 10.1039/D4FO03374D 39494504

